# At-sea distribution patterns of the Peruvian diving petrel *Pelecanoides garnotii* during breeding and non-breeding seasons

**DOI:** 10.1038/s41598-023-40975-z

**Published:** 2023-09-02

**Authors:** Claudia E. Fernández, Guillermo Luna-Jorquera, Cristián G. Suazo, Petra Quillfeldt

**Affiliations:** 1 Doctorado en Biología y Ecología Aplicada, Facultad de Ciencias del Mar, Universidad Católica del Norte, Larrondo 1281, Coquimbo, Chile; 2 Department of Marine Biology, Center for Ecology and Sustainable Management of Oceanic Islands ESMOI, Facultad de Ciencias del Mar, Universidad Católica del Norte, Larrondo 1281, Coquimbo, Chile; 3 Centro de Estudios Avanzados en Zonas Áridas (CEAZA), Coquimbo, Chile; 4 Department of Animal Ecology and Systematics, Justus Liebig University Giessen, Heinrich-Buff-Ring 26, 35392 Giessen, Germany

**Keywords:** Ecology, Ecology

## Abstract

At-sea distributions of seabird species are strongly associated with the distribution patterns of their prey, which are influenced by physical oceanic features. During breeding and non-breeding seasons, seabirds move extraordinary distances among different environments. However, foraging site fidelity by seabirds appears to be high in areas of known high productivity, such as frontal zones and upwellings. Here, we present a tracking study for the Peruvian diving-petrel *Pelecanoides garnotii*, an endemic seabird of the highly productive Humboldt Current System, to assess whether adults use the same foraging areas throughout the year, combining data from nest monitoring and global location sensors (GLS) deployed on 12 individuals between two breeding seasons (2013–2014 and 2014–2015), in Choros Island (29°15′S; 71°32′W), Chile. Two main foraging areas were registered. During the breeding season, adults moved in the northern direction, between 60 to 144 km away from their colony, foraging in areas with high primary productivity. During the non-breeding period, they moved to southern latitudes (~ 1200 km). Adults spent 37% and 63% of their time in flight/land and on/underwater activities, respectively. We determined that birds move northward from their colony during breeding, where prey availability seems more predictable throughout the year. However, during the non-breeding period, it is likely that other environmental factors influence the distribution pattern of the Peruvian diving-petrel.

## Introduction

Seabirds are conspicuous inhabitants of marine ecosystems, which spend ca. 90% of their time at sea and stay on land only for reproduction^1^. At-sea distributions for most seabirds are strongly associated with the distribution of their prey, which in turn is influenced by oceanographic features such as temperature and salinity^2,3^.

The life history traits of pelagic seabirds —such as low fecundity or small clutch sizes— are commonly presumed to be a consequence of the difficulty in obtaining energy at sea from patchy or widely scattered resources^4,5^. The hypothesis of foraging site fidelity^6^, posits that foraging site fidelity should be strongest when prey availability is predictable. This can be tested, by examining whether individuals return to the same site from one breeding season to the next, where high productivity at sea would be expected. Thus, in temperate and polar regions, seabirds appear to know the location and concentrations of patches at large and mesoscales^6,7^, in contrast to seabirds in tropical regions where the distribution of marine resources appears to be less predictable^6,8^.

During reproduction, seabirds are central-place foragers^9,10^. Foraging trips of breeding seabirds are constrained by the need to periodically return to the nest to incubate eggs or feed chicks^9,11^. In contrast, seabird inter-breeding movements can cover thousands of square kilometres, and individuals can visit different habitats and feeding conditions^12–14^. Procellariiformes (petrels, shearwaters, and albatrosses) are highly mobile seabirds that have evolved physiological and morphological adaptations, allowing them to exploit distant foraging areas efficiently^10,15^. During the non-breeding season, many species frequently occupy habitats markedly different from those used during breeding^16,17^. The diving-petrels, belonging to the family Procellariidae, are a genus (*Pelecanoides*) of five diving seabird species confined exclusively to the southern hemisphere. These species have wings with a high specialisation for diving^18^, an adaptation associated with limited flight capacity^14,18,19^, and in which movement patterns has been described^14,20–23^. Studies on the spatial distribution of Common diving-petrels *Pelecanoides urinatrix*, South Georgia diving-petrels *P*. *georgicus* and Whenua Hou diving-petrel *P. whenuahouensis* provided information about individual movements and foraging ecology during breeding^23–26^ and non-breeding periods^14,21,22,27^. However, little is known about the spatial patterns of Peruvian diving-petrels *Pelecanoides garnotii*^28^. Common, South Georgia, and Whenua Hou diving-petrels live in cold waters, where most of the breeding colonies are located around the Antarctic Polar Front and sub-Antarctic waters^22,26^, a harsh environment where individuals can breed only once a year^29,30^ when food availability increases during spring and summer^31^. Peruvian diving-petrels, in contrast, live in a less severe environment with cold and highly productive waters, the Humboldt Current System (HCS)^32^. In Peru, breeding occurs at least twice a year^33^; it seems that the oceanographic and climatic conditions have favoured this nesting strategy^32^.

It is well known that most of the Chilean coast is influenced by the HCS^34^. In this highly productive large marine ecosystem, upwelling-favourable winds occur year-round in the north (~ 26°S to 20°S), with a constant upwelling that sustains a very high primary productivity^32,34^, in contrast to more seasonal upwellings at southern latitudes such as ~ 35°S^32^. The Peruvian diving-petrel (referred to as "PDP" hereafter) is endemic in the HCS and has the most northerly distribution of all five species of diving-petrels^35^. During observational census or occasional records at-sea, PDPs have been registered in different seasons along the Chilean coast^36,37^. However, their spatial distribution during breeding and inter-breeding seasons is not well known. Here, we used the PDP as an ideal model organism to test the hypothesis of foraging site fidelity. Due to the influence of the HCS and the presence of a relatively permanent upwelling in the north (between 26°S and 20°S) of the main breeding colony of PDP in Chile (29°S, Choros Island), we expected that the PDPs would forage year-round in the same area where prey availability seems to be more predictable. In order to test this hypothesis, we used global location sensors (GLS) that are useful for studying the movements at large-scale of pelagic seabirds (with a spatial accuracy between 186 and 400 km^38,39^), and allowed us to obtain information about PDP movements during breeding and non-breeding seasons^9,14,20^.

## Results

**Figure 1:**
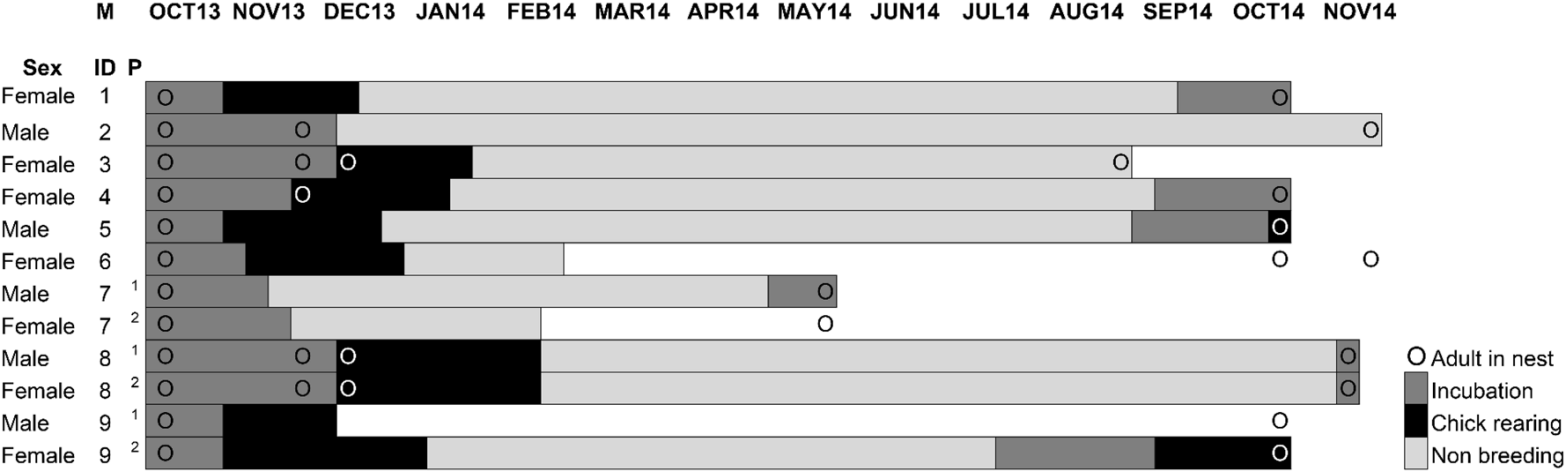
Chronology of the breeding (incubation, chick rearing) and non-breeding season for Peruvian diving-petrels during 2013–2014 and part of 2014–2015. GLS of individuals (denoted as ID) #3 and #7^1^ and #7^2^ were recovered in August and May, respectively. For individuals #6, #7^2^ and #9^1^, the tracking devices stopped working prematurely. Breeding pairs (P, two adults in the same nest) with devices correspond to those denoted with superscript 1 and 2. Presence of adults were detected on nests (circles) according to the monthly monitoring of nests.

Of the 20 Peruvian diving-petrels equipped with data loggers (from 17 nests), 12 (60%) adults were recaptured (Table 1). Two breeding pairs (one with both individuals equipped and one with one individual equipped) failed their breeding attempt during the first breeding season (Fig. 1). The first breeding season (October 2013) was recorded until the second week of February 2014 (Fig. 1). Some adults showed a breeding attempt during autumn (March–June) and winter months (June–September). In brief, the breeding pairs with GLS ID# 7^1^/7^2^ and 9^1^/9^2^ showed an incubation period during May (attempt failed) and July 2014 (successful attempt), respectively (Fig. 1). A second breeding season was recorded from September to November 2014.

**Table 1:** Date of deployment and recovery of global location sensors (GLS) in 20 breeding adults of Peruvian diving-petrels.

# Logger	Deployment of GLS (start date)	Recovery of GLS (end date)	Downloaded data successfully	Downloaded data partially	Bird ID code	Sex
1	24/10/2013	22/10/2014	+		1	Female
2	24/10/2013	22/11/2014	+		2	Male
22	22/10/2013	26/08/2014	+		3	Female
28	24/10/2013	23/10/2014	+		4	Female
43	22/10/2013	23/10/2014	+		5	Male
32	22/10/2013	22/10/2014		+	6	Female
23	21/10/2013	20/05/2014	+		7^1^	Male
42	22/10/2013	19/05/2014		+	7^2^	Female
34	21/10/2013	22/11/2014	+		8^1^	Male
38	22/10/2013	20/11/2014	+		8^2^	Female
37	21/10/2013	22/10/2014		+	9^1^	Male
44	22/10/2013	22/10/2014	+		9^2^	Female
3	22/10/2013	NR				
4	21/10/2013	NR				
21	22/10/2013	NR				
24	22/10/2013	NR				
26	21/10/2013	NR				
30	21/10/2013	NR				
36	22/10/2013	NR				
41	22/10/2013	NR				

Recovered GLS were identified with a code (1–9). Breeding pairs denoted with superscript: 7^1^/7^2^, 8^1^/8^2^ and 9^1^/9^2^. *NR* Not recovered.

**Figure 2:**
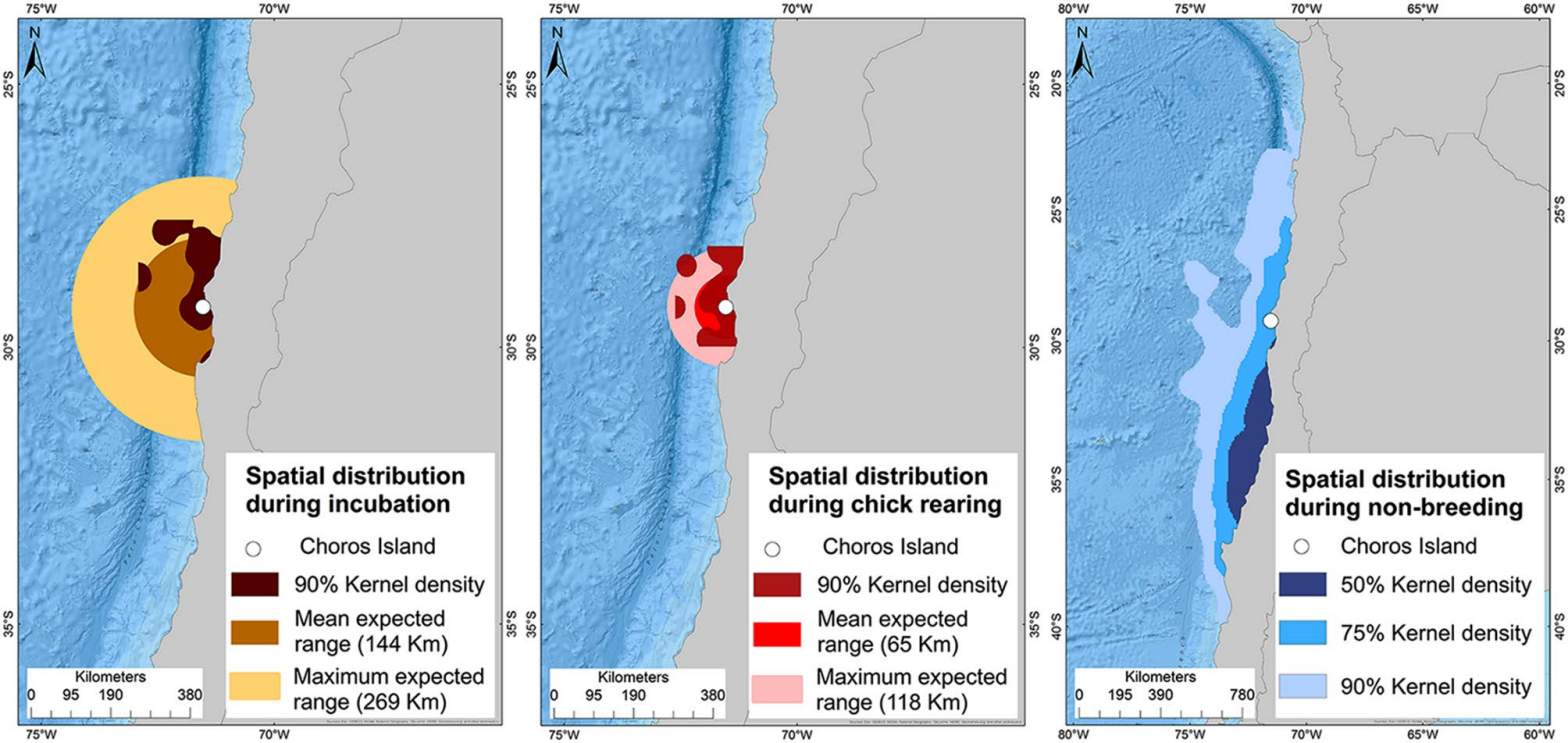
At-sea spatial distribution of Peruvian diving-petrels during breeding (incubation, chick rearing) and non-breeding seasons. Influence zones for incubation and chick rearing periods are shown for the mean expected range (dark color) and the maximum expected range (light color). Also, 90% kernel density areas are represented during the breeding period. During non-breeding, 50 and 90% kernel density areas are represented by darker and lighter tone contours, respectively. The map for the distribution of the non-breeding period presents a larger geographic scale than during the incubation and chick rearing period maps.

During breeding (i.e., incubation, chick rearing), most petrels moved northwards from Choros Island (Fig. S2 in the Supplementary information). Breeding males and females had similar distributions during 2013–2014 and 2014–2015. In the ocean, most individuals showed a mean longitudinal distribution between 70° and 71°W. According to method 1, the mean home distance of individuals during the incubation phase was 686 ± 459 km, and during chick rearing, it was 524 ± 331 km (Table S1 and Fig. S2 in the Supplementary information). The trip duration during the incubation and chick-rearing phases were 16 ± 3 h and 17 ± 2 h, respectively. According to method 2, with corrected home distance values, the mean home distance of individuals during the incubation phase was 144 ± 125 km, and during chick rearing, it was 65 ± 53 km. The estimated trip duration during the incubation and chick-rearing phases were 21 ± 6 h and 19 ± 3 h, respectively. Results obtained with method 2 showed that during the incubation and chick-rearing periods, an important spatial overlap occurred in the distribution of individuals according to 90% kernel densities (Fig. 2). The distribution of individuals in the breeding season was 27°S to 30°S. During non-breeding, individuals mainly ranged south of the reproductive colony with a mean distance of 634 ± 364 km. Overall, the distributional range of petrels at 90% kernel densities was 27°S to 40°S, which includes part of northern-central and central-southern Chile.

**Figure 3:**
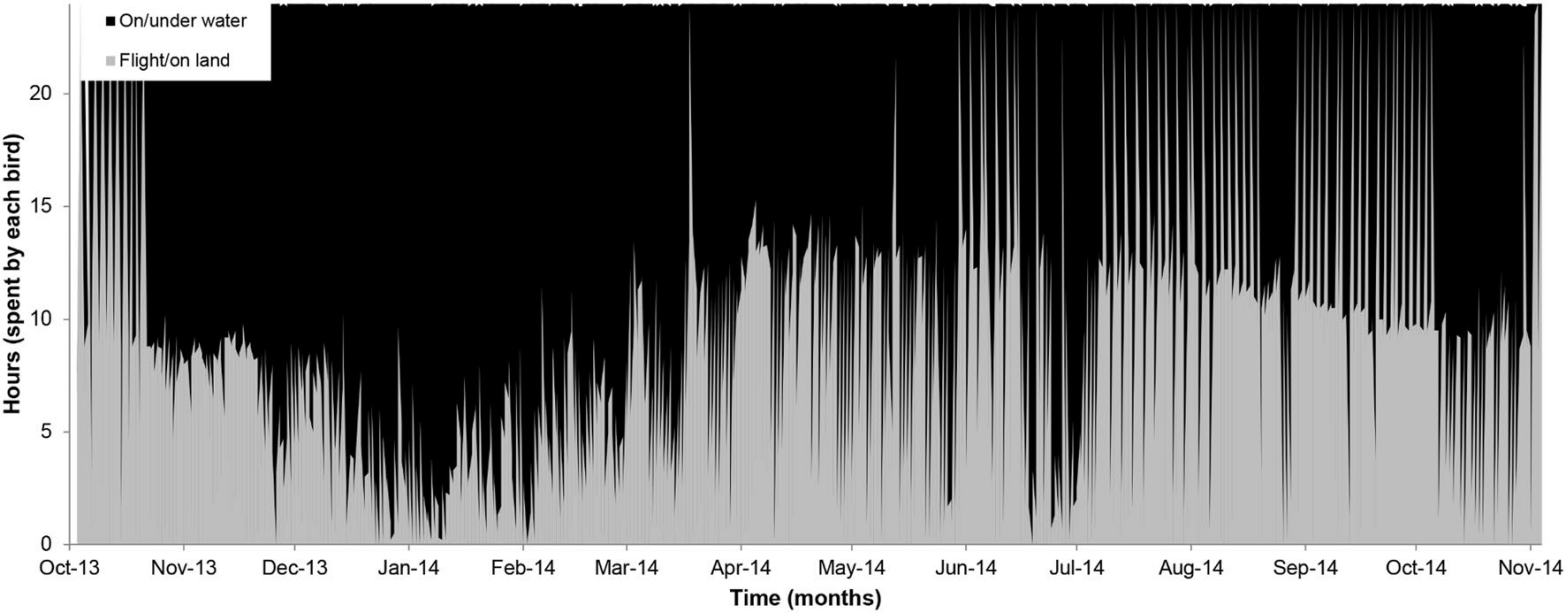
Time (h) spent in different activities, wet recordings (on/under water) and dry recordings (flight/land) of Peruvian diving-petrels throughout the study period.

**Figure 4:**
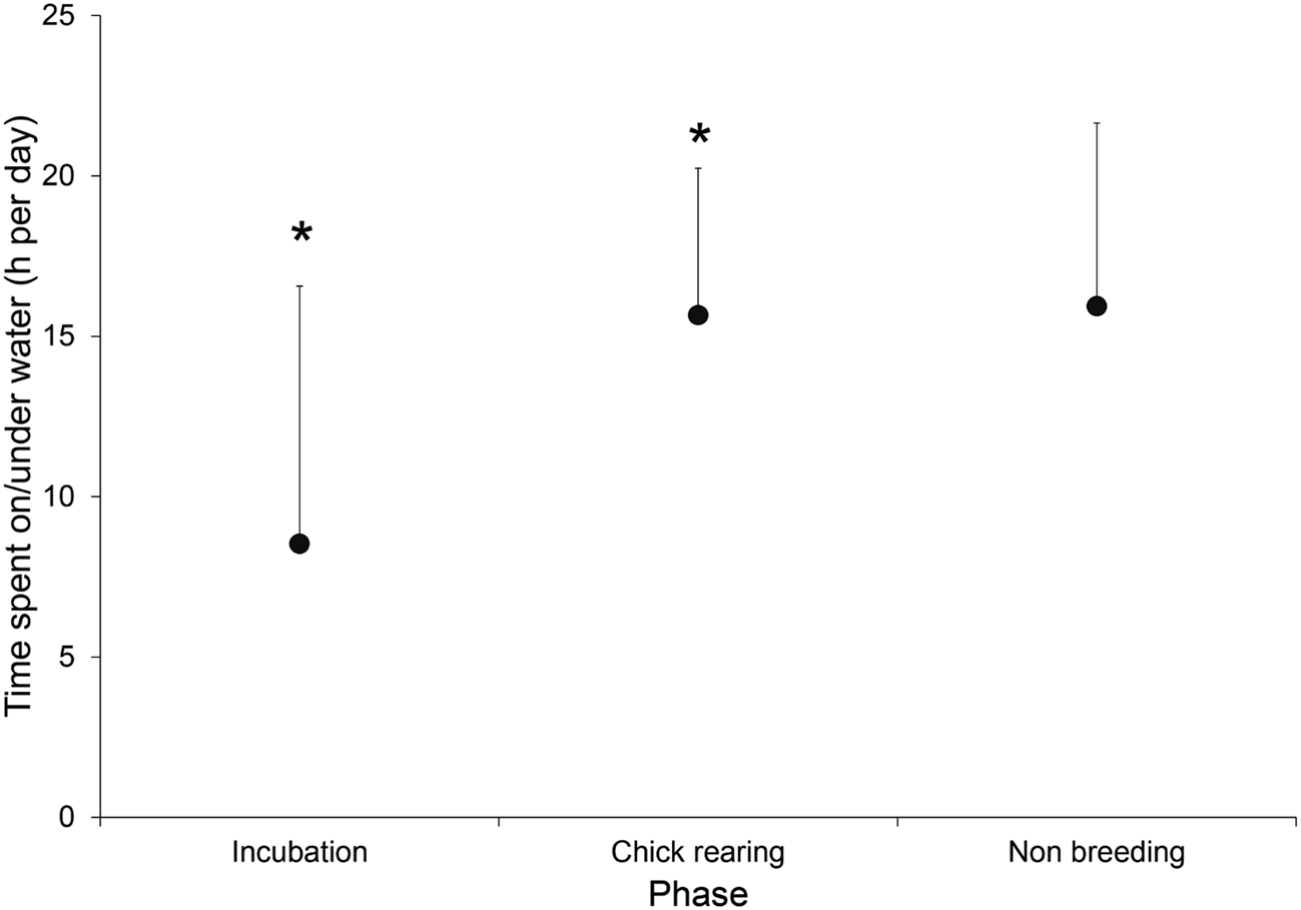
Mean values (± SD) of total time spent on/under water activity (h) in relation to seasons. Asterisk (*) show significant differences among groups (*P* = 0.05).

The time (mean h day^−1^ ± SD) spent by individuals at flight/land and on/under water were 9 ± 7 and 15 ± 9 h day^−1^, respectively. Among phases, flight/land activity mainly occurred during incubation (from October to November 2013 and September to October 2014), and on/under water activities frequently occurred during chick rearing and non-breeding (Table 2, Fig. 3). We observed a peak of time that individuals spent on/under water (Fig. 3) during January and February. Adults showed differences in the daily time used for on/under water activities among phases (Table 3). Significant differences were detected in the time on/under water per day between incubation and chick-rearing phases (*P* = 0.02, *P* value following Bonferroni correction, Fig. 4).

**Table 2:** Activity patterns of Peruvian diving-petrels during incubation, chick rearing and non-breeding phases.

Activity	Sex	Incubation		Chick rearing		Non-breeding
		Period I	Period II	Period I	Period II	
Wet recordings						
Time spent on/under water (h day^−1^)	Female	9 ± 8	8 ± 8	16 ± 5	14 ± 1	17 ± 6
	Male	8 ± 8	7 ± 7	15 ± 4	–	15 ± 5
Dry recordings						
Time spent flight/land (h day^−1^)	Female	15 ± 8	16 ± 8	8 ± 5	10 ± 1	7 ± 6
	Male	16 ± 8	17 ± 7	9 ± 4	–	9 ± 5

Mean values and standard deviation (SD) are given. Periods I and II include the reproductive season 2013–2014 and 2014–2015, respectively.

**Table 3:** Results of generalised linear mixed-effects models (GLMMs) testing the effect of phase, sex and the interaction of phase × sex in the time spent on/under water.

Dependent	Independent	*F*	*P*-value
Time spent on/under water (h)	Phase	3.90	**0.020**
	Sex	0.04	0.838
	Phase × sex	2.00	0.131
	Covariate		
	Flight/land		**˂ 0.001**

Flight/land is included as covariate. Significant p-values are marked in bold.

## Discussion

This study described new aspects of the at-sea spatial patterns of the Peruvian diving-petrels in the southern region of the HCS. Our main result showed two different foraging distributions differing between breeding and non-breeding seasons: PDPs move towards northern latitudes in the direction of the upwelling centre located relatively close to their colony during the breeding period and move to southern latitudes during the non-breeding period.

Most of the Chilean coast is influenced by the HCS^34^. The HCS is a highly productive large marine ecosystem, which generates upwelling centres in the northern (~ 23°S) and northern-central zones (~ 30°S) and is characterised to be mostly continuous, in contrast to more seasonal upwellings at latitudes south of ~ 35°S^32^. Two coastal upwelling centres have been described near Choros Island (~ 100 km); one of these centres is located to the South (Punta Lengua de Vaca, ~ 30.5°S) and the other to the north of the island (Punta de Choros, ~ 29°S)^40,41^. Overall, during both the 2013–2014 and 2014–2015 breeding seasons, birds from Choros Island consistently showed a northward distribution. This area is characterised by upwelling-favourable winds that sustain a very high primary productivity^32,42^. Our results thus suggest that breeding adults forage around this latter coastal upwelling centre (e.g., Punta de Choros), moving mainly in the northward direction from their colony, to exploit these predictable and abundant food resources.

Across their distributional range, the Peruvian diving-petrels feed on fish larvae and mainly euphausiids, particularly *Euphausia mucronata*^33,43^. This species is considered one of the most abundant species of the HCS, shows vertical migration, remaining in deeper waters (~ 250 m) during the day and ascending to the sea surface (0–50 m) before dusk^44–46^; however, euphausiid swarms are also found near the surface during daylight^47^. A high-density centre of *E. mucronata* has been described at ~ 30°S and 96 km near the coast^48^, and the presence of fin whales between 29′00°S and 29′20°S (around Choros and Chañaral Islands) were associated with a high concentration *E. mucronata*^47,49^. The local upwelling events, which are more frequent during spring and summer, make possible the availability of this high productivity. The distribution patterns of diving-petrels in Coquimbo Bay (~ 30°S) during summer were previously described using at-sea counts from a research vessel^50^, showing that high densities of individuals were directly south of Choros Island in an upwelling area. This matches our results, although we observed that adults prefer to move northward to the closest upwelling zone that is also exploited by fin whales feeding on the same prey^49^. Thus, food availability, because of upwelling systems near the colonies, plays a major role in determining the foraging strategy of PDPs during the breeding period. The latter is also consistent with the results found for South Georgian diving-petrels, which, during the chick-rearing period^23^, visit the same distant feeding areas every year because of a consistent and reliable food source.

We detected a remarkable inter-individual consistency during winter in the routes and areas selected for wintering. Also, our observations revealed that winter migration of PDPs to southern latitudes (~ 35°S) coincides with a reduction in primary productivity in those areas. These results are surprising since, as we mentioned above, upwelling centres of the HCS change from continuous to more seasonal from low to high latitudes. Thus, in central-southern Chile (at ~ 37°S), an important and seasonal upwelling event occurs during austral spring-summer^44^, and PDPs may take advantage of this during the early part of the non-breeding season, especially for their moult. However, during the austral autumn–winter months (March–September), upwelling at 37°S is weak or absent^44,51^, which implies a reduction in Chlorophyll-*a*. Although Chlorophyll-*a* is an indirect indicator widely used to determine the at-sea distribution of seabirds^52^, it seems unlikely that it is a good predictor for the distribution of PDPs during winter months. Still, food resources in wintering areas of the southern Chilean coast seem to be sufficient to cope with food demands and supply them with energy reserves for the next breeding attempt. Peruvian diving-petrels can shift prey choice seasonally, from euphausiids to larval stages of fish^43^, which allows them to change diet composition when the availability of marine resources varies throughout the year. The main prey, *E. mucronata*, shows no clear seasonal pattern throughout the year^44^ but seems to be able to adapt to changing environmental conditions and shows continuous growth year-round^45,53^. Moreover, although this krill species grow better in upwelling conditions, it is not necessarily limited when weak upwelling or downwelling occurs^45^. For PDPs, inter-annual differences in the quantity and quality of food resources in the winter grounds should be very important in determining the body condition of individuals for the next breeding attempt.

Foraging distances in seabirds are described for a growing number of species, particularly during breeding periods^20,54–56^. In the five extant species of diving-petrels, the relatively short wings specialised for diving^18^ is associated with limited flight capacity^18,19^ and, consequently, limited foraging distances^14^. Previous estimates of foraging distances (using GLS) for Common and South Georgia diving-petrels during incubation and non-breeding periods were about 260 and 3000 km, respectively^14,20^. Recently, for both species, the mean maximum distance from the colony was estimated, using GPS, to range 19–322 km during incubation^24–26^, and 19–217 km during chick rearing^23,25,26^. For the Whenua Hou diving-petrel, the mean maximum distance estimated (using GLS) from the colony during non-breeding was about 3700 km^22^. We found a mean maximum distance from the colony of 144 km during incubation and 65 km during the chick-rearing phase; both records are similar to those reported for the Common and South Georgia diving-petrels. Except for the non-breeding period, the mean distance found in this study (634 km) is slightly less than the reported for the other species of diving-petrels^14,21,22^. The reasons why PDPs do not fly further to the south have yet to be determined. Climatic factors during winter in southern Chile could set restrictions to the movements, although the PDP is endemic to the HCS that extends to ca. 42°S, and it is considered environmentally less severe than those faced by the Common, South Georgia, and Whenua Hou diving petrels in their marine habitat. In addition, by moving along the Humboldt Current System, the PDP likely find feeding grounds where food availability allows them to offset the cost of remaining at sea during the winter.

During the non-breeding period, dry recordings showed a high value (~ 9 h day^−1^). However, PDPs are unlikely to sustain continuous flight that would produce the observed dry pattern (Fig. 3), as we mentioned above, because of limited flight capabilities. As expected, they were not at the colony during this period (Fig. 1). Recently, a study using GLS in Common diving-petrels showed that individuals could spend ~ 44 min flying continuously during the post-breeding migration^27^. Although individuals could reach over 1000 km day^−1^ in the non-breeding period, long flight trips were restricted to only 5% of the time the birds spent in this activity (dry records)^27^. A study of the inter-breeding movements of little auks *Alle alle* (120–180 g, a diving species)^57^ showed similar results to our study. They observed that the daily dry recordings from the saltwater sensor never exceeded 50% (~ 9 h) of the time during the non-breeding period. However, they indicated that dry recordings are not necessarily equal to the proportion of time spent flying because birds could leave their legs in their plumage, or they could be sitting on sea ice. For whiskered auklets, *Aethia pygmea*, a species of small size (~ 111 g), a high number of dry recordings, particularly at night, was observed, which was associated with roosting behaviour on land^58^. In other seabird species, dry readings had also been associated with individuals that tuck their legs out of the water while afloat (e.g., Atlantic Puffins *Fratercula arctica*)^59^. Although PDP is, in general, distributed close to the coast, observations have not indicated that the species rests at night on the continent unless they are attracted by artificial lights, as happened in similar petrels^60^, and also it has been observed for PDPs during the breeding period (C. Fernández, pers. obs.). Then, for PDPs, the most plausible explanation is that animals tuck their legs in their plumage while afloat. It is important to consider that the use of immersion data for inferring activity patterns is limited in Peruvian diving-petrels; indeed, the method will not allow for identifying the time flying from when the birds are not flying.

GLS has been widely used in monitoring large-scale movements of many species^12,14,61,62^. Despite their low spatial accuracy of 186–400 km^38,39^ compared to other tracking devices (e.g., GPS, PTT), the low cost and mass of GLS allow monitoring small species and large sample sizes^61^. This study sheds light on the distribution of PDP during the breeding period. However, the interpretation of our results during this period should be viewed with caution because life history traits may affect interpretation. For example, diving seabird species, such as diving-petrels, may spend hours at the same foraging spot^24,63^ between multiple dives or sitting on the water^26^. Unlike surface feeder species with better flight characteristics, they can travel thousands of kilometres in search of different options for feeding grounds^64,65^. Our results suggest that dry recordings in PDPs can also be explained by the time that birds keep their legs in their plumage while sitting on the water. While for surface-feeding species, the interpretation of the GLS results would suggest that the time that the device is out of the water (dry recordings) may be explained as time spent flying. In addition, during the breeding season, PDPs usually fly daily, especially during the chick-rearing period, and GLS only register the time when birds are heading towards (sunshine) and coming back (sunset) from the foraging areas. Thus, considering the limitations of using GLS, we could translate the data into a conservative but realistic spatial distribution pattern for the PDP during the breeding season. High-resolution devices, such as miniaturised GPS, are required to better characterise the at-sea distribution patterns and home distance range during the breeding period of PDPs.

In summary, our year-round tracking research indicates that PDPs move towards northern latitudes in the direction of the upwelling centre located relatively close to their colony during the breeding period. In these upwelling centres, prey availability seems more predictable throughout the year. However, PDPs move to southern latitudes during the non-breeding period. The non-breeding season starts at the end of the Austral summer when the phytoplankton bloom might still be good in the South. Like other seabird species, the PDP travel from their nesting sites to distant places during the non-breeding period. In wintering areas, oceanographic conditions and food availability seem to influence the foraging behaviour of PDPs strongly. Multi-annual tracking studies, including high-resolution devices, could confirm whether PDPs' breeding and interbreeding movements will consistently follow the same foraging routes.

## Methods

### Study site and GLS attachment

Choros Island (29°15′S, 71°32′W, 322 ha) is located 6 km off the shore of Punta Choros, some 100 km north of Coquimbo, Chile. On this island is found ~ 90% of the breeding population of Peruvian diving-petrels in Chile^66^. A total of 20 GLS (Biotrack, Dorset, UK; MK5) were deployed during the breeding season 2013–2014 (Table 1). In total, twelve devices (60%) were recovered. The GLS weighed ~ 1 g (< 1% of the mean body mass, 220 ± 27 [SD] g, N = 46 of diving-petrels). As PDPs are burrow nesters, we first determined nest occupancy using a burrow scope camera before selecting a nest. In total, 17 nests with adults incubating eggs were marked and geo-referenced. In these nests, 20 adults were captured (six adults corresponded to reproductive pairs, Table 1) by introducing a hand into the burrows. Then, we took morphometric measurements: length of the bill (cm), head (cm), tarsus (cm), wing (cm), tail (cm), leg diameter (mm), and body mass (g). Additionally, we took blood samples for sex determination. GLS devices were attached to a plastic ring with a cable tie, and placed on the right leg of each individual. This procedure ensured that the GLS loggers remained on the animals for a year (Table 1). The procedure took about 10 min, and birds were returned to their breeding burrows immediately afterwards. This study was carried out in compliance with the ARRIVE guidelines. All relevant guidelines and regulations for the care and use of animals were followed, including animal ethics approvals from the Bioethics Committee of the Universidad Católica del Norte, Coquimbo, Chile (letter authorisation of 17 December 2014). Permits to capture, band, and handle birds were approved by the Servicio Agrícola y Ganadero of Chile (SAG) (Resolution N° 7238/2013). All field experimental protocols and access to Choros Island were authorised by the Corporación Nacional Forestal, CONAF (Letter N°62/2014).

All individuals from which we recovered GLS (N = 12, Table 1) were in the same nest where they were captured the first time. Individuals were weighed and measured upon recovery. The nests were examined monthly from October 2013 to December 2014. Monitoring consisted of determining the occupation of nests (i.e., presence of an adult, an adult with egg/chick, only chick) by using the endoscopic camera and then checking the presence of adults with GLS attached to their leg. Thus, by combining tracking data from GLS with information on monthly visits, we estimated in each nest the time (days) of adult spent incubating (27 ± 18 [SD] days) and chick-rearing (presence of chick in the nest, 56 ± 11 days) and the period of non-breeding (absence of adults in the nest, 212 ± 87 days). The duration of incubation and rearing period for PDPs has been reported to vary between 50–60 days and 70–80 days, respectively^33^. Here, it was not possible to record the incubation period from its beginning; in total, it was recorded between 10 to 45 days.

### Positional and immersion data processing from GLS

Light data were analysed with post-processing BAS-Track software (British Antarctic Survey, Cambridge, UK), followed by ‘TransEdit’ to check for integrity of light curves and to determine dawn and dusk times, and ‘Locator’ to estimate the latitude from day length and longitude from the time of local mid-day with respect to Greenwich Mean Time. We assumed a sun elevation angle of –3.0° based on known positions obtained during pre- and post-deployment calibration of the loggers at the colony. All estimated locations were examined visually in a geographic information system (GIS), and any unrealistic positions —either associated with interference to light curves at dawn or dusk or in proximity to equinoxes when latitudes are unreliable— were excluded from further analyses. GLS provide two positions per day based on light levels, with an accuracy of 186–400 km^38,39^. The deployment and recovery dates, the sex of the birds and other details are given in Table 1 and Fig. 1.

After an exhaustive re-examination of all estimated locations and considering the intrinsic errors in the positions obtained from the GLS devices, we still obtained several positions located over land areas, indicating a longitudinal error. However, the spatial distribution pattern during the breeding and non-breeding season was mainly found in latitudinal differences. PDPs are considered coastal foragers^67,68^ and have been seen foraging very close to the mainland (~ 10 m, C. Fernández, pers. obs.), which could also produce erroneous land positions.

To infer the birds' behaviour at sea, data were analysed using the online tool Actave.net^69^, also recently used to infer the behaviour at sea of the Whenua Hou diving-petrel^22^. The GLS recorded saltwater immersion every 3 s as a proxy for activity patterns and stored the sum of positive tests once every 10 min. Hence, each recorded time-stamped immersion value (denoted ε) can range from 0 (no immersion, continuously dry) to 200 (permanently immersed, continuously wet). Using Actave’s standard settings, we use immersion values to define cumulative counts as follows: (i) time in flight/land: the sum of all 10-min intervals with ε = 0 (dry) and (ii) time on/under water: the sum of 10-min intervals with 0 < ε ≤ 200 (wet). The parameters obtained from Actave.net are daily summary values, home distance (in km, which describes how far the bird was from the location of logger deployment), and activity types (in hours, in sum amounting to 24) as characterised above (i.e., flight/land, on/under water). For flight/land activity, it was not possible to separate flight from land activities during the breeding season because the saltwater immersion logger only detects “dry recordings” with an immersion value of zero. Then, the time spent by the birds on the land during the breeding period was calculated from the combination of nest monitoring and light and activity data, determining if dry recordings during the day coincided with the presence of the adult in the nest (Fig. 1). During the non-breeding period, most equipped Peruvian diving-petrels were exclusively at sea. Here, the high proportion of time spent in dry recordings may arise from activities other than flying (e.g., birds tuck their legs in their plumage). Thus, the total of dry recordings (flight/land activity) was not necessarily equal to the proportion of time spent flying continuously.

We used two methods to obtain the home distance and trip duration values for the breeding period of PDPs. First, we used the Actave.net program (method 1, described above, Table S1 in the Supplementary information). Second, we corrected the home distance values obtained from the Actave.net program (method 2, Table S1 in the Supplementary information). The correction was made because we considered that the home distance values were high during the reproductive period (incubation: mean 686 ± 459 km, N = 181, chick rearing: mean 524 ± 331 km, N = 479) even after outliers removal (incubation: mean 510 ± 350 km, N = 128, chick rearing: mean 479 ± 278 km, N = 452), particularly for a diving seabird species that is believed to have a limited flight capacity and is restricted to return to the colony to incubate or to feed chicks. Our criterion was supported by observations of the monthly monitoring of the nests and by the results of activity patterns (*flight/land*, *on/under water*) obtained from the Actave.net program, which showed that the time assigned to flight/land is restricted to 15 h day^−1^ during the incubation period and 8 h day^−1^ during chick rearing (Table 2, Table S2 in the Supplementary information). Therefore, we adjusted the home-distance values to the expected values using the flight speed of PDPs, ~ 45 km h^−1^ (C. Zavalaga, pers. Comm.). This flight speed value is similar to the average flight speed reported for South Georgian diving-petrels of 50 km h^−1^^23^. Afterwards, these results were processed in ArcGIS 10.3 (ESRI, Redlands, CA, USA) (see below). First, the time “within the nest” (on land) and the time “outside the nest” (at sea) were estimated for each individual (Table S2 in the Supplementary information), using the light and activity data and nest monitoring information. From the total recorded by the sensor (*S*), we subtracted the time of activity on/under water (w) [Eq. (1)]. Thus, a time of activity “flight” was estimated. Finally, the flight speed of PDPs (45 km h^−1^) was used to estimate the total distance travelled by day and then divided by two to obtain the expected home distance [Eq. (1)]. Zero values obtained after correction were not considered in subsequent analyses. The estimated trip duration was obtained by calculating the mean value of the time “outside the nest” (at sea) for each period (incubation, chick rearing).

1 $$\frac{\left(S-w\right)*\frac{45Km}{h}}{2}$$

For methods 1 and 2, we created influence zones around the breeding colony of PDPs (29°S, Choros Island) using the home distance values reached during the incubation and chick-rearing periods. Influence zones were plotted in ArcGIS 10.3. For method 1 (incubation: N = 181, chick rearing: N = 443), the influence zone included the mean and the maximum range (“Max R”) of home distance values (Fig. S1 in the Supplementary information). For method 2 (incubation: N = 160, chick rearing: N = 384), the influence zone included the mean expected range (“Mean Exp R”) and the maximum expected range (“Max Exp R”) of the home distance expected values (Fig. S1 in the Supplementary information). These influence zones act as proxies for the most distant areas of the colony (home distance) that the PDPs reached.

Changes in distribution among phases of the breeding and non-breeding seasons were examined using kernel analysis of filtered locations^38^. The non-parametric fixed kernel density estimator was used to determine density contours. Kernel densities do not require serial independence of observations when estimating foraging ranges^70^. Kernel analyses were performed in a WGS 1984 Web Mercator (auxiliary sphere) projection using ArcGIS 10.3. To perform the kernel density estimation for the incubation and chick-rearing periods, we considered the home distance values obtained from methods 1 and 2. In method 1, the data were not filtered (incubation: N = 181, chick rearing: N = 443). In method 2, the data were filtered; only those locations that did not exceed the expected maximum range of home distance (incubation: 269 km, N = 76 and chick rearing: 118 km, N = 36) were used. Following previous authors^71,72^, we used 90%, 75% and 50% kernel density contours to represent the foraging area (Fig. S2 in the Supplementary information).

The home distance values and the kernel analysis of individuals during the non-breeding period were not adjusted in this study. Our results are supported by the observations included in the eBird database. eBird is a broadscale bird monitoring project that collects observations made throughout the year by volunteers^73,74^. Participants follow a standardised checklist protocol, in which time, location, search effort, and the number of individuals of each species are all reported. To further improve data quality, eBird has expert volunteers who develop regional filters based on the chosen geographical coordinates and observations date^75^. Thus, we verified that the records we obtained from the eBird platform for the PDPs during the non-breeding period were in line with our results. For example, from 2007 to November 2017, about 15,325 observations of PDPs were recorded in Valparaíso (~ 33°S) during the autumn (March–June) and winter (June–September) months^37^.

### Molecular sexing

The sexes of adults were determined by DNA analyses using blood samples. DNA was extracted from FTA® Classic Cards using a DNeasy blood & tissue kit (Qiagen). The manufacturer’s protocol was followed. Sexing of birds is based on differences in length between introns in the CHD-Z and CHD-W genes^76^. For PCR, we used the primers developed by Fridolfsson and Ellegren (1999:2550F/2718R). All samples were run on a 1.5% agarose gel and checked for the presence of a single (male) or double (female) band. In total, 10 males and 10 females were identified (Table 1).

### Data analyses

All statistical procedures were performed with SYSTAT 12. The significance level used was P = 0.05. When necessary, data were transformed (ln [x + 1]) to meet the normality and homoscedasticity assumptions of parametric analysis^77^.

Generalised linear mixed models (GLMMs) were applied to test the effect of nesting phases (incubation, chick rearing, and non-breeding), sex, and its interaction on activity types, as described above. Bird identity (ID#) was included as a random factor to account for pseudo-replication issues. From activity types, we selected the wet recordings, namely on/under water activity (h), as the response variable because it is a critical activity for the survival of individuals. Flight/land activity (dry recordings) was used as a covariate because we considered that the amount of time (h) spent in one activity also depends on the amount of time spent on the other activity. When significant differences were detected among means, a post hoc test was performed using the sequential Bonferroni correction^78^, which adjusts the significance level according to the number of multiple comparisons.

### Supplementary Information

Supplementary Information.

## Data Availability

The datasets used and analysed are available from the corresponding author on reasonable request.
