# Supplementary material for: At-sea distribution patterns of the Peruvian diving petrel Pelecanoides garnotii during breeding and non-breeding seasons
Source: Sci Rep. 2023 Sep 2;13:14463. doi: 10.1038/s41598-023-40975-z (PMC10475040; doi:10.1038/s41598-023-40975-z)
Supplement: Supplementary file 1 — Supplementary Information. [file 41598_2023_40975_MOESM1_ESM.docx]

*Electronic Supplementary Material for Marine Biology research article:*

**At-sea distribution patterns of the Peruvian diving-petrel *Pelecanoides garnotii* during breeding and non-breeding seasons**

Claudia E. Fernández^1^, Guillermo Luna-Jorquera^*1,2,3^, Cristián G. Suazo^4^ & Petra Quillfeldt^4^

^1^Doctorado en Biología y Ecología Aplicada, Facultad de Ciencias del Mar, Universidad Católica del Norte, Larrondo 1281, Coquimbo, Chile

^2^Millenium Nucleus for Ecology and Sustainable Management of Oceanic Islands

^3^Centro de Estudios Avanzados en Zonas Áridas (CEAZA), Coquimbo, Chile

^4^Department of Animal Ecology and Systematics, Justus Liebig University Giessen, Heinrich-Buff-Ring 38, 35392 Giessen, Germany

*Corresponding author, E-mail: gluna@ucn.cl, +56512209816

**Table S1.** Homedistance values (km, obtained from Actave.net) versus maximum expected values (km, estimated values, see methods) for each adult equipped with data loggers during incubation and chick rearing phases.

| **Phase: Incubation** |  |  |  |  |
| --- | --- | --- | --- | --- |
| **Uniqueid** | **n** | **Homedistance (km)** | **Maximum expected range (km)** | **Expected values differ from observed values (%)** |
| 1 | 1 | 561 | 79 | 86 |
| 1 | 2 | 643 | 68 | 90 |
| 1 | 3 | 310 | 63 | 80 |
| 1 | 4 | 519 | 56 | 89 |
| 1 | 5 | 666 | 56 | 92 |
| 1 | 6 | 1163 | 52 | 96 |
| 1 | 7 | 957 | 45 | 95 |
| 1 | 8 | 948 | 0 | 100 |
| 2 | 9 | 423 | 41 | 90 |
| 2 | 10 | 344 | 41 | 88 |
| 2 | 11 | 506 | 34 | 93 |
| 2 | 12 | 237 | 29 | 88 |
| 2 | 13 | 225 | 29 | 87 |
| 2 | 14 | 22 | 29 | -33 |
| 2 | 15 | 402 | 23 | 94 |
| 2 | 16 | 696 | 23 | 97 |
| 2 | 17 | 1036 | 20 | 98 |
| 2 | 18 | 198 | 18 | 91 |
| 2 | 19 | 81 | 18 | 78 |
| 2 | 20 | 680 | 14 | 98 |
| 22 | 21 | 330 | 180 | 45 |
| 22 | 22 | 205 | 173 | 15 |
| 22 | 23 | 1158 | 173 | 85 |
| 22 | 24 | 208 | 173 | 17 |
| 22 | 25 | 37 | 173 | -368 |
| 22 | 26 | 928 | 171 | 82 |
| 22 | 27 | 635 | 169 | 73 |
| 22 | 28 | 145 | 167 | -15 |
| 22 | 29 | 780 | 164 | 79 |
| 22 | 30 | 242 | 162 | 33 |
| 22 | 31 | 192 | 162 | 16 |
| 22 | 32 | 643 | 162 | 75 |
|  |  |  |  | continue |
|  |  |  |  | continuation Table S1 |
| **Phase: Incubation** |  |  |  |  |
| **Uniqueid** | **n** | **Homedistance (km)** | **Maximum expected range (km)** | **Expected values differ from observed values (%)** |
| 22 | 33 | 314 | 162 | 48 |
| 22 | 34 | 188 | 158 | 16 |
| 22 | 35 | 184 | 153 | 17 |
| 22 | 36 | 773 | 151 | 80 |
| 22 | 37 | 727 | 151 | 79 |
| 22 | 38 | 204 | 151 | 26 |
| 22 | 39 | 262 | 140 | 47 |
| 22 | 40 | 1244 | 137 | 89 |
| 22 | 41 | 818 | 41 | 95 |
| 22 | 42 | 61 | 0 | 100 |
| 22 | 43 | 200 | 0 | 100 |
| 23 | 44 | 802 | 484 | 40 |
| 23 | 45 | 763 | 479 | 37 |
| 23 | 46 | 1056 | 477 | 55 |
| 23 | 47 | 430 | 466 | -8 |
| 23 | 48 | 800 | 461 | 42 |
| 23 | 49 | 656 | 461 | 30 |
| 23 | 50 | 476 | 461 | 3 |
| 23 | 51 | 126 | 459 | -264 |
| 23 | 52 | 276 | 450 | -63 |
| 23 | 53 | 467 | 360 | 23 |
| 23 | 54 | 834 | 311 | 63 |
| 23 | 55 | 1220 | 308 | 75 |
| 23 | 56 | 238 | 270 | -13 |
| 23 | 57 | 283 | 263 | 7 |
| 23 | 58 | 279 | 257 | 8 |
| 23 | 59 | 529 | 250 | 53 |
| 23 | 60 | 108 | 248 | -129 |
| 28 | 61 | 1090 | 108 | 90 |
| 28 | 62 | 1161 | 101 | 91 |
| 28 | 63 | 329 | 101 | 69 |
| 28 | 64 | 1064 | 99 | 91 |
| 28 | 65 | 1057 | 97 | 91 |
| 28 | 66 | 91 | 97 | -6 |
| 28 | 67 | 1023 | 97 | 91 |
|  |  |  |  | continue |
|  |  |  |  | continuation Table S1 |
| **Phase: Incubation** |  |  |  |  |
| **Uniqueid** | **n** | **Homedistance (km)** | **Maximum expected range (km)** | **Expected values differ from observed values (%)** |
| 28 | 68 | 896 | 97 | 89 |
| 28 | 69 | 258 | 95 | 63 |
| 28 | 70 | 507 | 95 | 81 |
| 28 | 71 | 1614 | 95 | 94 |
| 28 | 72 | 585 | 92 | 84 |
| 28 | 73 | 397 | 92 | 77 |
| 28 | 74 | 168 | 92 | 45 |
| 28 | 75 | 1441 | 92 | 94 |
| 28 | 76 | 1022 | 90 | 91 |
| 28 | 77 | 276 | 86 | 69 |
| 28 | 78 | 889 | 79 | 91 |
| 28 | 79 | 930 | 11 | 99 |
| 28 | 80 | 92 | 11 | 88 |
| 28 | 81 | 998 | 0 | 100 |
| 28 | 82 | 171 | 0 | 100 |
| 28 | 83 | 1304 | 0 | 100 |
| 32 | 84 | 1113 | 110 | 90 |
| 32 | 85 | 796 | 99 | 88 |
| 32 | 86 | 1470 | 95 | 94 |
| 32 | 87 | 1525 | 90 | 94 |
| 32 | 88 | 851 | 74 | 91 |
| 34 | 89 | 2036 | 128 | 94 |
| 34 | 90 | 2043 | 90 | 96 |
| 34 | 91 | 1339 | 79 | 94 |
| 34 | 92 | 937 | 70 | 93 |
| 34 | 93 | 202 | 70 | 65 |
| 34 | 94 | 1313 | 63 | 95 |
| 34 | 95 | 1409 | 63 | 96 |
| 34 | 96 | 741 | 63 | 91 |
| 34 | 97 | 939 | 63 | 93 |
| 34 | 98 | 727 | 59 | 92 |
| 34 | 99 | 1313 | 56 | 96 |
| 34 | 101 | 1272 | 56 | 96 |
| 34 | 102 | 819 | 56 | 93 |
| 34 | 100 | 1199 | 56 | 95 |
|  |  |  |  | continue |
|  |  |  |  | continuation Table S1 |
| **Phase: Incubation** |  |  |  |  |
| **Uniqueid** | **n** | **Homedistance (km)** | **Maximum expected range (km)** | **Expected values differ from observed values (%)** |
| 34 | 103 | 830 | 56 | 93 |
| 34 | 104 | 781 | 52 | 93 |
| 34 | 105 | 772 | 50 | 94 |
| 34 | 106 | 1326 | 50 | 96 |
| 34 | 107 | 906 | 45 | 95 |
| 34 | 108 | 174 | 45 | 74 |
| 34 | 109 | 819 | 41 | 95 |
| 34 | 110 | 898 | 36 | 96 |
| 34 | 111 | 16 | 36 | -125 |
| 34 | 112 | 199 | 34 | 83 |
| 34 | 113 | 709 | 29 | 96 |
| 34 | 114 | 847 | 0 | 100 |
| 34 | 115 | 1263 | 0 | 100 |
| 34 | 116 | 581 | 0 | 100 |
| 34 | 117 | 933 | 0 | 100 |
| 37 | 118 | 205 | 14 | 93 |
| 37 | 119 | 35 | 11 | 68 |
| 38 | 120 | 219 | 371 | -70 |
| 38 | 121 | 121 | 371 | -207 |
| 38 | 122 | 187 | 371 | -99 |
| 38 | 123 | 200 | 367 | -83 |
| 38 | 124 | 290 | 365 | -26 |
| 38 | 125 | 381 | 365 | 4 |
| 38 | 126 | 247 | 360 | -46 |
| 38 | 127 | 141 | 356 | -152 |
| 38 | 128 | 334 | 356 | -6 |
| 38 | 129 | 172 | 353 | -105 |
| 38 | 130 | 313 | 353 | -13 |
| 38 | 131 | 488 | 349 | 29 |
| 38 | 132 | 483 | 315 | 35 |
| 38 | 133 | 877 | 205 | 77 |
| 38 | 134 | 353 | 173 | 51 |
| 38 | 135 | 295 | 169 | 43 |
| 38 | 136 | 283 | 158 | 44 |
| 38 | 137 | 241 | 158 | 35 |
|  |  |  |  | continue |
|  |  |  |  | continuation Table S1 |
| **Phase: Incubation** |  |  |  |  |
| **Uniqueid** | **n** | **Homedistance (km)** | **Maximum expected range (km)** | **Expected values differ from observed values (%)** |
| 38 | 138 | 347 | 158 | 55 |
| 38 | 139 | 155 | 158 | -2 |
| 38 | 140 | 460 | 158 | 66 |
| 42 | 141 | 1562 | 191 | 88 |
| 42 | 142 | 1989 | 169 | 92 |
| 42 | 143 | 1588 | 169 | 89 |
| 42 | 144 | 1715 | 164 | 90 |
| 42 | 145 | 2059 | 153 | 93 |
| 42 | 146 | 1313 | 151 | 89 |
| 42 | 147 | 743 | 97 | 87 |
| 42 | 148 | 907 | 95 | 90 |
| 42 | 149 | 522 | 95 | 82 |
| 42 | 150 | 397 | 92 | 77 |
| 42 | 151 | 855 | 92 | 89 |
| 42 | 152 | 634 | 90 | 86 |
| 42 | 153 | 906 | 90 | 90 |
| 42 | 154 | 977 | 81 | 92 |
| 42 | 155 | 887 | 65 | 93 |
| 42 | 156 | 1610 | 38 | 98 |
| 42 | 157 | 594 | 0 | 100 |
| 43 | 158 | 1059 | 38 | 96 |
| 43 | 159 | 721 | 27 | 96 |
| 43 | 160 | 1042 | 11 | 99 |
| 43 | 161 | 1240 | 0 | 100 |
| 43 | 162 | 813 | 0 | 100 |
| 43 | 163 | 1550 | 0 | 100 |
| 43 | 164 | 680 | 0 | 100 |
| 43 | 165 | 607 | 0 | 100 |
| 43 | 166 | 1044 | 0 | 100 |
| 44 | 167 | 427 | 83 | 81 |
| 44 | 168 | 440 | 79 | 82 |
| 44 | 169 | 575 | 56 | 90 |
| 44 | 170 | 897 | 50 | 94 |
| 44 | 171 | 1058 | 45 | 96 |
| 44 | 172 | 193 | 45 | 77 |
|  |  |  |  | continue |
|  |  |  |  | continuation Table S1 |
| **Phase: Incubation** | |  |  |  |
| **Uniqueid** | **n** | **Homedistance (km)** | **Maximum expected range (km)** | **Expected values differ from observed values (%)** |
| 44 | 173 | 640 | 0 | 100 |
| 44 | 174 | 327 | 0 | 100 |
| 44 | 175 | 844 | 0 | 100 |
| 44 | 176 | 295 | 0 | 100 |
| 44 | 177 | 981 | 0 | 100 |
| 44 | 178 | 425 | 0 | 100 |
| 44 | 179 | 505 | 0 | 100 |
| 44 | 180 | 134 | 0 | 100 |
| 44 | 181 | 721 | 0 | 100 |
| **Phase: Chick rearing** | |  |  |  |
| **Uniqueid** | **n** | **Homedistance (km)** | **Maximum expected range (km)** | **Expected values differ from observed values (%)** |
| 1 | 1 | 565 | 72 | 87 |
| 1 | 2 | 625 | 56 | 91 |
| 1 | 3 | 791 | 52 | 93 |
| 1 | 4 | 479 | 52 | 89 |
| 1 | 5 | 160 | 50 | 69 |
| 1 | 6 | 956 | 50 | 95 |
| 1 | 7 | 320 | 50 | 85 |
| 1 | 8 | 719 | 50 | 93 |
| 1 | 9 | 1065 | 47 | 96 |
| 1 | 10 | 288 | 47 | 84 |
| 1 | 11 | 303 | 45 | 85 |
| 1 | 12 | 399 | 45 | 89 |
| 1 | 13 | 215 | 45 | 79 |
| 1 | 14 | 641 | 43 | 93 |
| 1 | 15 | 677 | 43 | 94 |
| 1 | 16 | 760 | 43 | 94 |
| 1 | 17 | 659 | 41 | 94 |
| 1 | 18 | 700 | 41 | 94 |
| 1 | 19 | 417 | 36 | 91 |
| 1 | 20 | 476 | 34 | 93 |
| 1 | 21 | 594 | 34 | 94 |
| 1 | 22 | 320 | 32 | 90 |
| 1 | 23 | 554 | 29 | 95 |
| 1 | 24 | 189 | 29 | 85 |
|  |  |  |  | continue |
|  |  |  |  | continuation Table S1 |
| **Phase: Chick rearing** | |  |  |  |
| **Uniqueid** | **n** | **Homedistance (km)** | **Maximum expected range (km)** | **Expected values differ from observed values (%)** |
| 1 | 25 | 497 | 27 | 95 |
| 1 | 26 | 276 | 27 | 90 |
| 1 | 27 | 265 | 27 | 90 |
| 1 | 28 | 343 | 27 | 92 |
| 1 | 29 | 252 | 25 | 90 |
| 1 | 30 | 348 | 23 | 94 |
| 1 | 31 | 587 | 23 | 96 |
| 1 | 32 | 501 | 20 | 96 |
| 1 | 33 | 190 | 0 | 100 |
| 1 | 34 | 146 | 0 | 100 |
| 1 | 35 | 173 | 0 | 100 |
| 1 | 36 | 515 | 0 | 100 |
| 1 | 37 | 741 | 0 | 100 |
| 1 | 38 | 484 | 0 | 100 |
| 1 | 39 | 511 | 0 | 100 |
| 1 | 40 | 588 | 0 | 100 |
| 1 | 41 | 188 | 0 | 100 |
| 1 | 42 | 581 | 0 | 100 |
| 1 | 43 | 373 | 0 | 100 |
| 22 | 44 | 613 | 135 | 78 |
| 22 | 45 | 792 | 113 | 86 |
| 22 | 46 | 453 | 113 | 75 |
| 22 | 47 | 931 | 104 | 89 |
| 22 | 48 | 1316 | 97 | 93 |
| 22 | 49 | 263 | 88 | 67 |
| 22 | 50 | 287 | 88 | 69 |
| 22 | 51 | 672 | 79 | 88 |
| 22 | 52 | 652 | 77 | 88 |
| 22 | 53 | 247 | 74 | 70 |
| 22 | 54 | 806 | 74 | 91 |
| 22 | 55 | 846 | 70 | 92 |
| 22 | 56 | 345 | 70 | 80 |
| 22 | 57 | 352 | 70 | 80 |
| 22 | 58 | 320 | 70 | 78 |
| 22 | 59 | 334 | 65 | 80 |
| 22 | 60 | 149 | 63 | 58 |
|  |  |  |  | continue |
|  |  |  |  | continuation Table S1 |
| **Phase: Chick rearing** | |  |  |  |
| **Uniqueid** | **n** | **Homedistance (km)** | **Maximum expected range (km)** | **Expected values differ from observed values (%)** |
| 22 | 61 | 208 | 61 | 71 |
| 22 | 62 | 406 | 56 | 86 |
| 22 | 63 | 326 | 52 | 84 |
| 22 | 64 | 268 | 47 | 82 |
| 22 | 65 | 782 | 43 | 95 |
| 22 | 66 | 350 | 34 | 90 |
| 22 | 67 | 476 | 25 | 95 |
| 22 | 68 | 305 | 18 | 94 |
| 22 | 69 | 190 | 4 | 98 |
| 22 | 70 | 879 | 0 | 100 |
| 22 | 71 | 239 | 0 | 100 |
| 22 | 72 | 1072 | 0 | 100 |
| 22 | 73 | 372 | 0 | 100 |
| 22 | 74 | 190 | 0 | 100 |
| 22 | 75 | 258 | 0 | 100 |
| 22 | 76 | 242 | 0 | 100 |
| 28 | 77 | 189 | 61 | 68 |
| 28 | 78 | 1140 | 59 | 95 |
| 28 | 79 | 761 | 59 | 92 |
| 28 | 80 | 403 | 56 | 86 |
| 28 | 81 | 641 | 56 | 91 |
| 28 | 82 | 777 | 56 | 93 |
| 28 | 83 | 741 | 56 | 92 |
| 28 | 84 | 158 | 56 | 64 |
| 28 | 85 | 229 | 56 | 75 |
| 28 | 86 | 265 | 56 | 79 |
| 28 | 87 | 414 | 56 | 86 |
| 28 | 88 | 261 | 56 | 78 |
| 28 | 89 | 920 | 52 | 94 |
| 28 | 90 | 293 | 52 | 82 |
| 28 | 91 | 450 | 52 | 89 |
| 28 | 93 | 955 | 50 | 95 |
| 28 | 94 | 212 | 47 | 78 |
| 28 | 95 | 579 | 47 | 92 |
| 28 | 96 | 456 | 45 | 90 |
| 28 | 97 | 589 | 45 | 92 |
|  |  |  |  | continue |
|  |  |  |  | continuation Table S1 |
| **Phase: Chick rearing** | |  |  |  |
| **Uniqueid** | **n** | **Homedistance (km)** | **Maximum expected range (km)** | **Expected values differ from observed values (%)** |
| 28 | 92 | 22 | 50 | -125 |
| 28 | 98 | 87 | 45 | 48 |
| 28 | 99 | 573 | 45 | 92 |
| 28 | 100 | 156 | 43 | 73 |
| 28 | 101 | 611 | 43 | 93 |
| 28 | 102 | 818 | 43 | 95 |
| 28 | 103 | 241 | 43 | 82 |
| 28 | 104 | 538 | 41 | 92 |
| 28 | 105 | 339 | 41 | 88 |
| 28 | 106 | 806 | 36 | 96 |
| 28 | 107 | 152 | 34 | 78 |
| 28 | 108 | 471 | 34 | 93 |
| 28 | 109 | 510 | 34 | 93 |
| 28 | 110 | 119 | 32 | 74 |
| 28 | 111 | 517 | 29 | 94 |
| 28 | 112 | 81 | 29 | 64 |
| 28 | 113 | 94 | 18 | 81 |
| 28 | 114 | 380 | 18 | 95 |
| 28 | 115 | 663 | 16 | 98 |
| 28 | 116 | 425 | 16 | 96 |
| 28 | 117 | 852 | 16 | 98 |
| 28 | 118 | 457 | 14 | 97 |
| 28 | 119 | 269 | 11 | 96 |
| 28 | 120 | 333 | 0 | 100 |
| 28 | 121 | 297 | 0 | 100 |
| 28 | 122 | 228 | 0 | 100 |
| 28 | 123 | 99 | 0 | 100 |
| 28 | 124 | 412 | 0 | 100 |
| 28 | 125 | 640 | 0 | 100 |
| 28 | 126 | 353 | 0 | 100 |
| 32 | 127 | 709 | 140 | 80 |
| 32 | 128 | 693 | 135 | 81 |
| 32 | 129 | 900 | 131 | 86 |
| 32 | 130 | 449 | 128 | 71 |
| 32 | 131 | 249 | 124 | 50 |
| 32 | 132 | 1024 | 124 | 88 |
|  |  |  |  | continue |
|  |  |  |  | continuation Table S1 |
| **Phase: Chick rearing** | |  |  |  |
| **Uniqueid** | **n** | **Homedistance (km)** | **Maximum expected range (km)** | **Expected values differ from observed values (%)** |
| 32 | 133 | 522 | 124 | 76 |
| 32 | 134 | 402 | 122 | 70 |
| 32 | 135 | 708 | 119 | 83 |
| 32 | 136 | 734 | 119 | 84 |
| 32 | 137 | 1143 | 119 | 90 |
| 32 | 138 | 1185 | 119 | 90 |
| 32 | 139 | 1028 | 119 | 88 |
| 32 | 140 | 1145 | 119 | 90 |
| 32 | 141 | 803 | 119 | 85 |
| 32 | 142 | 746 | 117 | 84 |
| 32 | 143 | 271 | 117 | 57 |
| 32 | 144 | 264 | 117 | 56 |
| 32 | 145 | 1263 | 117 | 91 |
| 32 | 146 | 494 | 115 | 77 |
| 32 | 147 | 378 | 113 | 70 |
| 32 | 148 | 516 | 113 | 78 |
| 32 | 149 | 549 | 113 | 80 |
| 32 | 150 | 729 | 113 | 85 |
| 32 | 151 | 488 | 108 | 78 |
| 32 | 152 | 532 | 108 | 80 |
| 32 | 153 | 198 | 101 | 49 |
| 32 | 154 | 476 | 95 | 80 |
| 32 | 155 | 971 | 72 | 93 |
| 32 | 156 | 1301 | 68 | 95 |
| 32 | 157 | 1049 | 68 | 94 |
| 32 | 158 | 1397 | 65 | 95 |
| 32 | 159 | 776 | 61 | 92 |
| 32 | 160 | 559 | 56 | 90 |
| 32 | 161 | 756 | 50 | 93 |
| 32 | 162 | 389 | 45 | 88 |
| 32 | 163 | 464 | 34 | 93 |
| 32 | 164 | 127 | 34 | 73 |
| 32 | 165 | 1085 | 23 | 98 |
| 32 | 166 | 649 | 11 | 98 |
| 32 | 167 | 250 | 11 | 96 |
| 32 | 168 | 593 | 4 | 99 |
|  |  |  |  | continue |
|  |  |  |  | continuation Table S1 |
| **Phase: Chick rearing** | |  |  |  |
| **Uniqueid** | **n** | **Homedistance (km)** | **Maximum expected range (km)** | **Expected values differ from observed values (%)** |
| 32 | 169 | 563 | 0 | 100 |
| 32 | 170 | 642 | 0 | 100 |
| 32 | 171 | 985 | 0 | 100 |
| 32 | 172 | 873 | 0 | 100 |
| 32 | 173 | 274 | 0 | 100 |
| 32 | 174 | 426 | 0 | 100 |
| 32 | 175 | 920 | 0 | 100 |
| 32 | 176 | 1197 | 0 | 100 |
| 32 | 177 | 321 | 0 | 100 |
| 32 | 178 | 491 | 0 | 100 |
| 32 | 179 | 839 | 0 | 100 |
| 32 | 180 | 1146 | 0 | 100 |
| 32 | 181 | 96 | 0 | 100 |
| 32 | 182 | 1045 | 0 | 100 |
| 32 | 183 | 297 | 0 | 100 |
| 34 | 184 | 1149 | 95 | 92 |
| 34 | 185 | 533 | 86 | 84 |
| 34 | 186 | 568 | 83 | 85 |
| 34 | 187 | 686 | 81 | 88 |
| 34 | 188 | 780 | 79 | 90 |
| 34 | 189 | 746 | 79 | 89 |
| 34 | 190 | 730 | 68 | 91 |
| 34 | 191 | 633 | 63 | 90 |
| 34 | 192 | 619 | 61 | 90 |
| 34 | 193 | 879 | 61 | 93 |
| 34 | 194 | 571 | 61 | 89 |
| 34 | 195 | 1220 | 56 | 95 |
| 34 | 196 | 1157 | 56 | 95 |
| 34 | 197 | 954 | 56 | 94 |
| 34 | 198 | 785 | 56 | 93 |
| 34 | 199 | 395 | 56 | 86 |
| 34 | 200 | 1018 | 54 | 95 |
| 34 | 201 | 797 | 54 | 93 |
| 34 | 202 | 995 | 52 | 95 |
| 34 | 203 | 669 | 52 | 92 |
| 34 | 204 | 788 | 50 | 94 |
|  |  |  |  | continue |
|  |  |  |  | continuation Table S1 |
| **Phase: Chick rearing** | |  |  |  |
| **Uniqueid** | **n** | **Homedistance (km)** | **Maximum expected range (km)** | **Expected values differ from observed values (%)** |
| 34 | 205 | 628 | 50 | 92 |
| 34 | 206 | 849 | 50 | 94 |
| 34 | 207 | 1042 | 50 | 95 |
| 34 | 208 | 576 | 50 | 91 |
| 34 | 209 | 565 | 50 | 91 |
| 34 | 210 | 653 | 47 | 93 |
| 34 | 211 | 526 | 47 | 91 |
| 34 | 212 | 532 | 47 | 91 |
| 34 | 213 | 689 | 45 | 93 |
| 34 | 214 | 1544 | 45 | 97 |
| 34 | 215 | 738 | 45 | 94 |
| 34 | 216 | 707 | 45 | 94 |
| 34 | 217 | 590 | 45 | 92 |
| 34 | 218 | 1175 | 43 | 96 |
| 34 | 219 | 594 | 43 | 93 |
| 34 | 220 | 711 | 43 | 94 |
| 34 | 221 | 588 | 41 | 93 |
| 34 | 222 | 1370 | 41 | 97 |
| 34 | 223 | 595 | 41 | 93 |
| 34 | 224 | 924 | 41 | 96 |
| 34 | 225 | 592 | 38 | 94 |
| 34 | 226 | 1014 | 38 | 96 |
| 34 | 227 | 1343 | 36 | 97 |
| 34 | 228 | 968 | 34 | 97 |
| 34 | 229 | 683 | 34 | 95 |
| 34 | 230 | 842 | 34 | 96 |
| 34 | 231 | 810 | 29 | 96 |
| 34 | 232 | 570 | 23 | 96 |
| 34 | 233 | 527 | 14 | 97 |
| 34 | 234 | 721 | 7 | 99 |
| 34 | 235 | 785 | 0 | 100 |
| 34 | 236 | 743 | 0 | 100 |
| 34 | 237 | 726 | 0 | 100 |
| 34 | 238 | 505 | 0 | 100 |
| 34 | 239 | 611 | 0 | 100 |
| 34 | 240 | 1005 | 0 | 100 |
|  |  |  |  | continue |
|  |  |  |  | continuation Table S1 |
| **Phase: Chick rearing** | |  |  |  |
| **Uniqueid** | **n** | **Homedistance (km)** | **Maximum expected range (km)** | **Expected values differ from observed values (%)** |
| 34 | 241 | 532 | 0 | 100 |
| 34 | 242 | 720 | 0 | 100 |
| 34 | 243 | 802 | 0 | 100 |
| 34 | 244 | 712 | 0 | 100 |
| 34 | 245 | 796 | 0 | 100 |
| 37 | 246 | 163 | 43 | 74 |
| 37 | 247 | 253 | 34 | 87 |
| 37 | 248 | 236 | 32 | 87 |
| 37 | 249 | 408 | 29 | 93 |
| 37 | 250 | 277 | 27 | 90 |
| 37 | 251 | 184 | 25 | 87 |
| 37 | 252 | 238 | 23 | 91 |
| 37 | 253 | 254 | 20 | 92 |
| 37 | 254 | 58 | 16 | 73 |
| 37 | 255 | 224 | 16 | 93 |
| 37 | 256 | 206 | 16 | 92 |
| 37 | 257 | 339 | 14 | 96 |
| 37 | 258 | 128 | 11 | 91 |
| 37 | 259 | 176 | 11 | 94 |
| 37 | 260 | 142 | 11 | 92 |
| 37 | 261 | 265 | 7 | 97 |
| 37 | 262 | 99 | 7 | 93 |
| 37 | 263 | 165 | 7 | 96 |
| 37 | 264 | 370 | 7 | 98 |
| 37 | 265 | 131 | 4 | 97 |
| 37 | 266 | 43 | 4 | 90 |
| 37 | 267 | 307 | 4 | 99 |
| 37 | 268 | 120 | 4 | 96 |
| 37 | 269 | 36 | 0 | 100 |
| 37 | 270 | 119 | 0 | 100 |
| 37 | 271 | 135 | 0 | 100 |
| 37 | 272 | 381 | 0 | 100 |
| 37 | 273 | 119 | 0 | 100 |
| 37 | 274 | 86 | 0 | 100 |
| 37 | 275 | 340 | 0 | 100 |
| 37 | 276 | 203 | 0 | 100 |
|  |  |  |  | continue |
|  |  |  |  | continuation Table S1 |
| **Phase: Chick rearing** | |  |  |  |
| **Uniqueid** | **n** | **Homedistance (km)** | **Maximum expected range (km)** | **Expected values differ from observed values (%)** |
| 37 | 277 | 52 | 0 | 100 |
| 37 | 278 | 79 | 0 | 100 |
| 37 | 279 | 191 | 0 | 100 |
| 38 | 280 | 364 | 230 | 37 |
| 38 | 281 | 444 | 230 | 48 |
| 38 | 282 | 123 | 230 | -87 |
| 38 | 283 | 90 | 225 | -150 |
| 38 | 284 | 496 | 223 | 55 |
| 38 | 285 | 238 | 212 | 11 |
| 38 | 286 | 540 | 209 | 61 |
| 38 | 287 | 111 | 209 | -89 |
| 38 | 288 | 159 | 209 | -32 |
| 38 | 289 | 139 | 207 | -49 |
| 38 | 290 | 171 | 207 | -21 |
| 38 | 291 | 275 | 207 | 25 |
| 38 | 292 | 88 | 203 | -130 |
| 38 | 293 | 18 | 203 | -1025 |
| 38 | 294 | 587 | 198 | 66 |
| 38 | 295 | 587 | 198 | 66 |
| 38 | 296 | 406 | 196 | 52 |
| 38 | 297 | 199 | 196 | 2 |
| 38 | 298 | 513 | 194 | 62 |
| 38 | 299 | 129 | 194 | -50 |
| 38 | 300 | 574 | 191 | 67 |
| 38 | 301 | 272 | 191 | 30 |
| 38 | 302 | 20 | 191 | -856 |
| 38 | 303 | 193 | 191 | 1 |
| 38 | 304 | 462 | 189 | 59 |
| 38 | 305 | 49 | 187 | -281 |
| 38 | 306 | 108 | 187 | -73 |
| 38 | 307 | 170 | 185 | -9 |
| 38 | 308 | 99 | 180 | -82 |
| 38 | 309 | 241 | 178 | 26 |
| 38 | 310 | 459 | 176 | 62 |
| 38 | 311 | 199 | 176 | 12 |
| 38 | 312 | 84 | 176 | -109 |
|  |  |  |  | continue |
|  |  |  |  | continuation Table S1 |
| **Phase: Chick rearing** | |  |  |  |
| **Uniqueid** | **n** | **Homedistance (km)** | **Maximum expected range (km)** | **Expected values differ from observed values (%)** |
| 38 | 313 | 213 | 173 | 19 |
| 38 | 314 | 163 | 160 | 2 |
| 38 | 315 | 318 | 158 | 50 |
| 38 | 316 | 354 | 151 | 57 |
| 38 | 317 | 477 | 149 | 69 |
| 38 | 318 | 180 | 146 | 19 |
| 38 | 319 | 471 | 146 | 69 |
| 38 | 320 | 414 | 142 | 66 |
| 38 | 321 | 94 | 140 | -48 |
| 38 | 322 | 420 | 135 | 68 |
| 38 | 323 | 371 | 128 | 65 |
| 38 | 324 | 182 | 117 | 36 |
| 38 | 325 | 228 | 117 | 49 |
| 38 | 326 | 198 | 113 | 43 |
| 38 | 327 | 143 | 106 | 26 |
| 38 | 328 | 588 | 104 | 82 |
| 38 | 329 | 118 | 97 | 18 |
| 38 | 330 | 226 | 97 | 57 |
| 38 | 331 | 259 | 90 | 65 |
| 38 | 332 | 196 | 83 | 58 |
| 38 | 333 | 68 | 61 | 11 |
| 38 | 334 | 394 | 61 | 85 |
| 38 | 335 | 309 | 47 | 85 |
| 38 | 336 | 224 | 45 | 80 |
| 38 | 337 | 140 | 45 | 68 |
| 38 | 338 | 427 | 41 | 91 |
| 38 | 339 | 166 | 29 | 82 |
| 38 | 340 | 128 | 27 | 79 |
| 38 | 341 | 334 | 23 | 93 |
| 43 | 342 | 381 | 68 | 82 |
| 43 | 343 | 602 | 56 | 91 |
| 43 | 344 | 466 | 45 | 90 |
| 43 | 345 | 841 | 43 | 95 |
| 43 | 346 | 790 | 41 | 95 |
| 43 | 347 | 597 | 34 | 94 |
| 43 | 348 | 344 | 34 | 90 |
|  |  |  |  | continue |
|  |  |  |  | continuation Table S1 |
| **Phase: Chick rearing** | |  |  |  |
| **Uniqueid** | **n** | **Homedistance (km)** | **Maximum expected range (km)** | **Expected values differ from observed values (%)** |
| 43 | 349 | 194 | 34 | 83 |
| 43 | 350 | 277 | 34 | 88 |
| 43 | 351 | 336 | 34 | 90 |
| 43 | 352 | 733 | 32 | 96 |
| 43 | 353 | 777 | 32 | 96 |
| 43 | 354 | 364 | 29 | 92 |
| 43 | 355 | 363 | 29 | 92 |
| 43 | 356 | 843 | 27 | 97 |
| 43 | 357 | 560 | 27 | 95 |
| 43 | 358 | 467 | 27 | 94 |
| 43 | 359 | 643 | 25 | 96 |
| 43 | 360 | 362 | 23 | 94 |
| 43 | 361 | 471 | 23 | 95 |
| 43 | 362 | 453 | 23 | 95 |
| 43 | 363 | 402 | 23 | 94 |
| 43 | 364 | 636 | 20 | 97 |
| 43 | 365 | 677 | 18 | 97 |
| 43 | 366 | 257 | 18 | 93 |
| 43 | 367 | 742 | 18 | 98 |
| 43 | 368 | 475 | 18 | 96 |
| 43 | 369 | 828 | 16 | 98 |
| 43 | 370 | 1045 | 16 | 98 |
| 43 | 371 | 1125 | 16 | 99 |
| 43 | 372 | 1336 | 16 | 99 |
| 43 | 373 | 787 | 14 | 98 |
| 43 | 374 | 380 | 14 | 96 |
| 43 | 375 | 401 | 11 | 97 |
| 43 | 376 | 333 | 11 | 97 |
| 43 | 377 | 326 | 7 | 98 |
| 43 | 378 | 806 | 7 | 99 |
| 43 | 379 | 755 | 4 | 99 |
| 43 | 380 | 687 | 2 | 100 |
| 43 | 381 | 694 | 0 | 100 |
| 43 | 382 | 989 | 0 | 100 |
| 43 | 383 | 86 | 0 | 100 |
| 43 | 384 | 229 | 0 | 100 |
|  |  |  |  | continue |
|  |  |  |  | continuation Table S1 |
| **Phase: Chick rearing** | |  |  |  |
| **Uniqueid** | **n** | **Homedistance (km)** | **Maximum expected range (km)** | **Expected values differ from observed values (%)** |
| 43 | 385 | 381 | 0 | 100 |
| 43 | 386 | 467 | 0 | 100 |
| 43 | 387 | 614 | 0 | 100 |
| 43 | 388 | 658 | 0 | 100 |
| 43 | 389 | 388 | 0 | 100 |
| 43 | 390 | 330 | 0 | 100 |
| 43 | 391 | 774 | 0 | 100 |
| 43 | 392 | 336 | 0 | 100 |
| 44 | 393 | 971 | 95 | 90 |
| 44 | 394 | 227 | 79 | 65 |
| 44 | 395 | 1567 | 79 | 95 |
| 44 | 396 | 1189 | 77 | 94 |
| 44 | 397 | 242 | 77 | 68 |
| 44 | 398 | 1655 | 74 | 96 |
| 44 | 399 | 1779 | 74 | 96 |
| 44 | 400 | 647 | 70 | 89 |
| 44 | 401 | 1097 | 70 | 94 |
| 44 | 402 | 456 | 68 | 85 |
| 44 | 403 | 585 | 65 | 89 |
| 44 | 404 | 802 | 65 | 92 |
| 44 | 405 | 1315 | 65 | 95 |
| 44 | 406 | 253 | 63 | 75 |
| 44 | 407 | 595 | 61 | 90 |
| 44 | 408 | 917 | 59 | 94 |
| 44 | 409 | 1459 | 59 | 96 |
| 44 | 410 | 1020 | 59 | 94 |
| 44 | 411 | 946 | 56 | 94 |
| 44 | 412 | 1218 | 56 | 95 |
| 44 | 413 | 1000 | 54 | 95 |
| 44 | 414 | 1158 | 52 | 96 |
| 44 | 415 | 1081 | 52 | 95 |
| 44 | 416 | 1289 | 52 | 96 |
| 44 | 417 | 1653 | 52 | 97 |
| 44 | 418 | 813 | 47 | 94 |
| 44 | 419 | 225 | 45 | 80 |
| 44 | 420 | 814 | 45 | 94 |
|  |  |  |  | continue |
|  |  |  |  | continuation Table S1 |
| **Phase: Chick rearing** | |  |  |  |
| **Uniqueid** | **n** | **Homedistance (km)** | **Maximum expected range (km)** | **Expected values differ from observed values (%)** |
| 44 | 421 | 1163 | 45 | 96 |
| 44 | 422 | 111 | 45 | 59 |
| 44 | 423 | 429 | 45 | 90 |
| 44 | 424 | 1244 | 43 | 97 |
| 44 | 425 | 890 | 41 | 95 |
| 44 | 426 | 151 | 41 | 73 |
| 44 | 427 | 486 | 41 | 92 |
| 44 | 428 | 335 | 41 | 88 |
| 44 | 429 | 153 | 38 | 75 |
| 44 | 430 | 1022 | 38 | 96 |
| 44 | 431 | 191 | 38 | 80 |
| 44 | 432 | 396 | 38 | 90 |
| 44 | 433 | 437 | 38 | 91 |
| 44 | 434 | 252 | 36 | 86 |
| 44 | 435 | 340 | 34 | 90 |
| 44 | 436 | 959 | 34 | 96 |
| 44 | 437 | 613 | 34 | 94 |
| 44 | 438 | 447 | 34 | 92 |
| 44 | 439 | 333 | 34 | 90 |
| 44 | 440 | 559 | 34 | 94 |
| 44 | 441 | 152 | 34 | 78 |
| 44 | 442 | 558 | 34 | 94 |
| 44 | 443 | 507 | 34 | 93 |
| 44 | 444 | 515 | 32 | 94 |
| 44 | 445 | 243 | 32 | 87 |
| 44 | 446 | 226 | 29 | 87 |
| 44 | 447 | 867 | 29 | 97 |
| 44 | 448 | 545 | 27 | 95 |
| 44 | 449 | 449 | 25 | 94 |
| 44 | 450 | 391 | 25 | 94 |
| 44 | 451 | 367 | 23 | 94 |
| 44 | 452 | 404 | 23 | 94 |
| 44 | 453 | 458 | 18 | 96 |
| 44 | 454 | 23 | 16 | 32 |
| 44 | 455 | 609 | 16 | 97 |
| 44 | 456 | 290 | 14 | 95 |
|  |  |  |  | continue |
|  |  |  |  | continuation Table S1 |
| **Phase: Chick rearing** | |  |  |  |
| **Uniqueid** | **n** | **Homedistance (km)** | **Maximum expected range (km)** | **Expected values differ from observed values (%)** |
| 44 | 457 | 464 | 11 | 98 |
| 44 | 458 | 346 | 4 | 99 |
| 44 | 459 | 505 | 0 | 100 |
| 44 | 460 | 376 | 0 | 100 |
| 44 | 461 | 924 | 0 | 100 |
| 44 | 462 | 631 | 0 | 100 |
| 44 | 463 | 414 | 0 | 100 |
| 44 | 464 | 194 | 0 | 100 |
| 44 | 465 | 208 | 0 | 100 |
| 44 | 466 | 543 | 0 | 100 |
| 44 | 467 | 266 | 0 | 100 |
| 44 | 468 | 479 | 0 | 100 |
| 44 | 469 | 329 | 0 | 100 |
| 44 | 470 | 893 | 0 | 100 |
| 44 | 471 | 141 | 0 | 100 |
| 44 | 472 | 328 | 0 | 100 |
| 44 | 473 | 448 | 0 | 100 |
| 44 | 474 | 138 | 0 | 100 |
| 44 | 475 | 365 | 0 | 100 |
| 44 | 476 | 384 | 0 | 100 |
| 44 | 477 | 306 | 0 | 100 |
| 44 | 478 | 501 | 0 | 100 |
| 44 | 479 | 180 | 0 | 100 |

**Table S2** Estimated time (h) spent inside the nest (“Nest-In”, on land) or spent out the nest (“Nest-Out”, at sea) by each adult breeder of Peruvian diving-petrels during the incubation and chick rearing phases

|  | Incubation phase | | | | | | | | | | | |
| --- | --- | --- | --- | --- | --- | --- | --- | --- | --- | --- | --- | --- |
|  | Nest-In | Nest-Out | Nest-In | Nest-Out | Nest-In | Nest-Out | Nest-In | Nest-Out | Nest-In | Nest-Out | Nest-In | Nest-Out |
| #GLS | #01 | #01 | #02 | #02 | #03 | #03 | #04 | #04 | #05 | #05 | #06 | #06 |
|  | 11 | 23 | 19 | 14 | 40 | 16 | 10 | 24 | 41 | 14 | 40 | 16 |
|  | 33 | 15 | 29 | 14 | 33 | 15 | 33 | 14 | 34 | 14 | 33 | 16 |
|  | 33 | 15 | 43 | 15 | 33 | 15 | 33 | 15 | 34 | 14 | 32 | 18 |
|  | 33 | 15 | 33 | 15 | 57 | 16 | 33 | 15 | 34 | 14 | 30 | 32 |
|  |  |  | 33 | 15 | 32 | 17 | 33 | 15 | 33 |  | 33 | 15 |
|  |  |  | 57 | 15 | 31 | 17 | 33 | 15 |  |  | 33 |  |
|  |  |  | 33 | 15 | 31 | 16 | 33 | 15 |  |  |  |  |
|  |  |  | 33 | 15 | 32 | 19 | 33 | 15 |  |  |  |  |
|  |  |  | 9 | 15 | 29 | 20 | 33 | 19 |  |  |  |  |
|  |  |  | 57 | 39 | 52 | 45 | 29 | 40 |  |  |  |  |
|  |  |  | 33 | 15 | 51 | 47 | 53 | 19 |  |  |  |  |
|  |  |  | 33 | 15 | 49 | 23 | 53 | 19 |  |  |  |  |
|  |  |  | 33 | 16 | 25 | 41 | 32 | 17 |  |  |  |  |
|  |  |  | 32 | 16 | 55 | 17 | 31 |  |  |  |  |  |
|  |  |  | 32 | 16 | 32 | 18 |  |  |  |  |  |  |
|  |  |  | 32 | 15 | 30 | 18 |  |  |  |  |  |  |
|  |  |  | 57 | 16 | 30 | 17 |  |  |  |  |  |  |
|  |  |  | 8 | 16 | 31 | 19 |  |  |  |  |  |  |
|  |  |  | 8 | 16 | 29 |  |  |  |  |  |  |  |
| Mean (h) | 28 | 17 | 32 | 16 | 37 | 22 | 34 | 19 | 35 | 14 | 34 | 19 |
| Total (h) | 110 | 67 | 614 | 313 | 702 | 396 | 472 | 242 | 176 | 56 | 201 | 97 |
| Mean (hd^-1^) | 15 | 9 | 16 | 8 | 15 | 9 | 16 | 8 | 18 | 6 | 16 | 8 |
| continue | | | | | | | | | | | | |

| continuation Table S2 | | | | | | | | | | | | |
| --- | --- | --- | --- | --- | --- | --- | --- | --- | --- | --- | --- | --- |
|  | Incubation phase | | | | | | | | | | | |
|  | Nest-In | Nest-Out | Nest-In | Nest-Out | Nest-In | Nest-Out | Nest-In | Nest-Out | Nest-In | Nest-Out | Nest-In | Nest-Out |
| #GLS | #07^2^ | #07^2^ | #07^1^ | #07^1^ | #08^1^ | #08^1^ | #08^2^ | #08^2^ | #09^1^ | #09^1^ | #09^2^ | #09^2^ |
|  | 14 | 15 | 10 | 17 | 12 | 14 | 37 | 39 | 15 | 14 | 20 | 16 |
|  | 33 | 39 | 57 | 15 | 10 | 14 | 81 | 39 | 33 | 15 | 33 | 15 |
|  | 33 | 39 | 57 | 15 | 58 | 14 | 57 | 15 | 33 | 15 | 32 | 15 |
|  | 33 | 12 | 33 | 15 | 10 | 14 | 57 | 40 | 33 | 14 | 33 | 15 |
|  | 33 | 39 | 33 | 15 | 10 | 14 | 56 | 17 |  |  |  |  |
|  | 33 | 15 | 9 | 15 | 57 | 15 | 55 | 44 |  |  |  |  |
|  | 33 | 40 | 33 | 15 | 9 | 15 | 98 | 66 |  |  |  |  |
|  | 32 | 41 | 33 | 15 | 33 | 14 | 32 | 40 |  |  |  |  |
|  | 55 | 68 | 9 | 15 | 9 | 15 | 32 | 17 |  |  |  |  |
|  | 27 | 40 | 57 | 40 | 57 | 15 | 31 | 18 |  |  |  |  |
|  | 32 |  | 56 | 16 | 9 | 15 | 30 | 16 |  |  |  |  |
|  |  |  | 8 | 19 | 33 | 15 | 56 | 16 |  |  |  |  |
|  |  |  | 52 | 40 | 9 | 15 | 32 |  |  |  |  |  |
|  |  |  | 8 | 16 | 57 | 64 |  |  |  |  |  |  |
|  |  |  | 8 | 15 | 57 | 20 |  |  |  |  |  |  |
|  |  |  |  |  | 9 | 15 |  |  |  |  |  |  |
|  |  |  |  |  | 56 | 15 |  |  |  |  |  |  |
|  |  |  |  |  | 7 | 17 |  |  |  |  |  |  |
|  |  |  |  |  | 8 | 16 |  |  |  |  |  |  |
|  |  |  |  |  | 32 | 16 |  |  |  |  |  |  |
|  |  |  |  |  | 32 | 15 |  |  |  |  |  |  |
|  |  |  |  |  | 8 | 15 |  |  |  |  |  |  |
|  |  |  |  |  | 32 | 15 |  |  |  |  |  |  |
|  |  |  |  |  | 32 | 16 |  |  |  |  |  |  |
| Mean (h) | 33 | 35 | 31 | 19 | 27 | 17 | 50 | 31 | 29 | 15 | 30 | 15 |
| Total (h) | 358 | 348 | 463 | 283 | 646 | 413 | 654 | 367 | 114 | 58 | 118 | 61 |
| Mean (hd^-1^) | 12 | 12 | 15 | 9 | 15 | 9 | 15 | 9 | 16 | 8 | 16 | 9 |
| continue | | | | | | | | | | | | |

| continuation Table S2 | | | | | | | | | | |
| --- | --- | --- | --- | --- | --- | --- | --- | --- | --- | --- |
| Chick rearing phase | | | | | | | | | | |
|  | Nest-In | Nest-Out | Nest-In | Nest-Out | Nest-In | Nest-Out | Nest-In | Nest-Out | Nest-In | Nest-Out |
| #GLS | #01 | #01 | #03 | #03 | #04 | #04 | #05 | #05 | #06 | #06 |
|  | 33 | 15 | 29 | 18 | 32 | 16 | 9 | 15 | 33 | 15 |
|  | 33 | 15 | 28 | 20 | 32 | 16 | 9 | 14 | 9 | 15 |
|  | 33 | 19 | 29 | 19 | 32 | 16 | 10 | 14 | 8 | 16 |
|  | 4 | 15 | 28 | 20 | 9 | 16 | 10 | 14 | 9 | 15 |
|  | 33 | 15 | 25 | 23 | 9 | 15 | 9 | 14 | 33 | 15 |
|  | 9 | 15 | 24 | 24 | 8 | 15 | 9 | 15 | 6 | 18 |
|  | 9 | 15 | 7 | 17 | 9 | 15 | 8 | 16 | 8 | 16 |
|  | 9 | 15 | 31 | 17 | 8 | 15 | 9 | 14 | 9 | 15 |
|  | 9 | 15 | 6 | 19 | 8 | 15 | 9 | 14 | 4 | 20 |
|  | 9 | 15 | 7 | 17 | 8 | 16 | 9 | 15 | 4 | 20 |
|  | 9 | 15 | 4 | 20 | 9 | 15 | 9 | 15 | 9 | 15 |
|  | 8 | 15 | 4 | 20 | 8 | 16 | 9 | 15 | 6 | 66 |
|  | 9 | 15 | 5 | 19 | 8 | 16 | 9 | 15 | 4 | 20 |
|  | 8 | 16 | 7 | 17 | 8 | 16 | 9 | 15 | 8 | 16 |
|  | 8 | 16 | 4 | 20 | 8 | 16 | 9 | 15 | 8 | 16 |
|  | 8 | 16 | 6 | 18 | 8 | 16 | 8 | 16 | 8 | 16 |
|  | 5 | 18 | 6 | 18 | 8 | 16 | 8 | 16 | 9 | 15 |
|  | 9 | 15 | 7 | 41 | 8 | 16 | 8 | 15 | 8 | 16 |
|  | 9 | 15 | 6 | 18 | 8 | 16 | 8 | 16 | 8 | 16 |
|  | 8 | 16 | 8 | 17 | 8 | 16 | 7 | 17 | 8 | 16 |
|  | 8 | 16 | 7 | 17 | 7 | 17 | 9 | 15 | 8 | 15 |
|  | 6 | 18 | 7 | 18 | 8 | 16 | 9 | 15 | 8 | 16 |
|  | 6 | 17 | 7 | 18 | 8 | 16 | 9 | 15 | 8 | 16 |
|  | 8 | 16 | 7 | 17 | 8 | 16 | 7 | 16 | 8 | 15 |
|  | 8 | 16 | 6 | 17 | 8 | 16 | 9 | 17 | 8 | 16 |
| continue | | | | | | | | | | |

| continuation Table S2 | | | | | | | | | | |
| --- | --- | --- | --- | --- | --- | --- | --- | --- | --- | --- |
| Chick rearing phase | | | | | | | | | | |
|  | Nest-In | Nest-Out | Nest-In | Nest-Out | Nest-In | Nest-Out | Nest-In | Nest-Out | Nest-In | Nest-Out |
| #GLS | #01 | #01 | #03 | #03 | #04 | #04 | #05 | #05 | #06 | #06 |
|  | 5 | 19 | 4 | 20 | 7 | 17 | 8 | 15 | 8 | 16 |
|  | 8 | 16 | 4 | 20 | 8 | 16 | 9 | 16 | 8 | 16 |
|  | 8 | 16 | 5 | 44 | 8 | 16 | 9 | 15 | 8 | 16 |
|  | 8 | 16 | 5 | 20 | 7 | 17 | 8 | 16 | 8 | 16 |
|  | 8 | 16 | 3 | 20 | 8 | 16 | 8 | 16 | 8 | 16 |
|  | 8 | 16 | 4 |  | 7 | 16 | 8 | 16 | 8 | 16 |
|  | 8 | 16 |  |  | 8 | 41 | 9 | 15 | 6 | 18 |
|  | 8 | 16 |  |  | 8 | 16 | 9 | 15 | 5 | 19 |
|  | 7 | 17 |  |  | 5 | 18 | 8 | 16 | 6 | 18 |
|  | 7 | 17 |  |  | 7 | 19 | 8 | 16 | 6 | 18 |
|  | 8 | 16 |  |  | 6 | 17 | 8 | 16 | 5 | 19 |
|  | 4 | 44 |  |  | 7 | 19 | 8 | 16 | 3 | 19 |
|  | 5 |  |  |  | 3 | 17 | 8 | 16 | 3 | 24 |
|  |  |  |  |  | 7 | 21 | 7 | 17 | 8 | 64 |
|  |  |  |  |  | 6 | 18 | 8 | 16 | 4 | 40 |
|  |  |  |  |  | 7 | 17 | 7 | 17 | 2 | 21 |
|  |  |  |  |  | 2 | 17 | 7 | 18 | 6 | 48 |
|  |  |  |  |  | 4 | 46 | 4 | 20 | 6 | 65 |
|  |  |  |  |  | 7 | 36 | 3 | 22 | 7 |  |
|  |  |  |  |  | 7 | 16 | 2 |  |  |  |
|  |  |  |  |  | 8 | 17 |  |  |  |  |
|  |  |  |  |  | 7 | 16 |  |  |  |  |
| Mean (h) | 10 | 17 | 11 | 20 | 9 | 18 | 8 | 16 | 8 | 21 |
| Total (h) | 390 | 619 | 330 | 613 | 419 | 844 | 364 | 692 | 354 | 924 |
| Mean (hd^-1^) | 9 | 15 | 8 | 16 | 8 | 16 | 8 | 16 | 7 | 17 |
| continue | | | | | | | | | | |

| continuation Table S2 | | | | | | | | |
| --- | --- | --- | --- | --- | --- | --- | --- | --- |
| Chick rearing phase | | | | | | | | |
|  | Nest-In | Nest-Out | Nest-In | Nest-Out | Nest-In | Nest-Out | Nest-In | Nest-Out |
| #GLS | #08^1^ | #08^1^ | #08^2^ | #08^2^ | #09^1^ | #09^1^ | #09^2^ | #09^2^ |
|  | 32 | 16 | 32 | 16 | 33 | 15 | 33 | 15 |
|  | 32 | 16 | 32 | 16 | 33 | 15 | 25 | 15 |
|  | 32 | 16 | 31 | 17 | 9 | 15 | 28 | 23 |
|  | 32 | 16 | 32 | 16 | 33 | 15 | 9 | 20 |
|  | 32 | 21 | 28 | 19 | 8 | 16 | 33 | 15 |
|  | 26 | 20 | 28 | 43 | 9 | 15 | 32 | 15 |
|  | 4 | 16 | 23 | 23 | 9 | 15 | 9 | 15 |
|  | 8 | 16 | 7 | 20 | 9 | 15 | 8 | 17 |
|  | 8 | 16 | 8 | 16 | 9 | 15 | 8 | 16 |
|  | 8 | 16 | 4 | 16 | 8 | 15 | 7 | 17 |
|  | 8 | 16 | 3 | 16 | 8 | 16 | 6 | 18 |
|  | 8 | 16 | 8 | 17 | 9 | 15 | 6 | 18 |
|  | 8 | 16 | 7 | 41 | 9 | 15 | 5 | 19 |
|  | 8 | 19 | 7 | 17 | 8 | 16 | 6 | 18 |
|  | 5 | 16 | 7 | 65 | 8 | 16 | 9 | 15 |
|  | 8 | 17 | 8 | 17 | 8 | 16 | 8 | 16 |
|  | 7 | 16 | 7 | 16 | 8 | 16 | 8 | 16 |
|  | 8 | 17 | 8 | 20 | 8 | 16 | 8 | 15 |
|  | 7 | 16 | 7 | 17 | 8 | 16 | 8 | 16 |
|  | 8 | 16 | 8 | 16 | 8 | 16 | 9 | 15 |
|  | 7 | 16 | 6 | 18 | 8 | 16 | 8 | 15 |
|  | 8 | 16 | 6 | 18 | 8 | 16 | 8 | 15 |
|  | 8 | 16 | 7 | 17 | 8 | 15 | 8 | 16 |
|  | 8 | 40 | 5 | 19 | 7 | 16 | 8 | 16 |
|  | 8 | 17 | 4 | 44 | 7 | 17 | 8 | 16 |
| continue | | | | | | | | |
| continuation Table S2 | | | | | | | | |
| Chick rearing phase | | | | | | | | |
|  | Nest-In | Nest-Out | Nest-In | Nest-Out | Nest-In | Nest-Out | Nest-In | Nest-Out |
| #GLS | #08^1^ | #08^1^ | #08^2^ | #08^2^ | #09^1^ | #09^1^ | #09^2^ | #09^2^ |
|  | 7 | 16 | 3 | 20 | 8 | 17 | 8 | 16 |
|  | 7 | 16 | 5 | 43 | 7 | 16 | 8 | 16 |
|  | 8 | 16 | 4 | 20 | 8 | 17 | 8 | 16 |
|  | 8 | 65 | 3 | 137 | 9 | 16 | 8 | 15 |
|  | 7 | 16 | 6 | 94 | 8 | 16 | 8 | 16 |
|  | 8 | 16 | 8 | 16 | 8 | 16 | 8 | 16 |
|  | 8 | 16 | 8 | 16 | 8 | 16 | 8 | 16 |
|  | 8 | 16 | 8 | 16 | 8 | 16 | 8 | 16 |
|  | 7 | 18 | 8 | 16 | 8 | 16 | 8 | 16 |
|  | 5 | 16 | 5 | 16 | 8 | 16 | 8 | 16 |
|  | 8 | 20 | 5 | 22 | 8 | 16 | 6 | 18 |
|  | 4 | 16 | 8 | 16 | 8 | 17 | 6 | 17 |
|  | 8 | 16 | 8 | 16 | 8 | 16 | 3 | 17 |
|  | 8 | 40 | 8 | 16 | 8 | 16 | 3 |  |
|  | 8 | 16 | 7 | 16 | 7 | 17 | 6 | 18 |
|  | 8 | 16 | 7 | 17 | 8 | 17 | 4 | 20 |
|  | 8 | 16 | 7 | 17 | 5 | 19 | 2 | 22 |
|  | 8 | 16 | 8 | 16 | 6 | 18 | 2 | 27 |
|  | 8 | 20 | 6 | 17 | 6 | 16 | 4 | 42 |
|  | 8 | 21 | 5 | 19 | 2 |  | 8 | 16 |
|  | 8 | 16 | 6 | 18 | 7 | 18 |  |  |
|  | 3 | 16 | 6 | 18 |  |  |  |  |
|  | 6 | 17 | 4 | 19 |  |  |  |  |
|  | 8 | 20 |  |  |  |  |  |  |
|  | 8 | 16 |  |  |  |  |  |  |
| continue | | | | | | | | |
| continuation Table S2 | | | | | | | | |
| Chick rearing phase | | | | | | | | |
|  | Nest-In | Nest-Out | Nest-In | Nest-Out | Nest-In | Nest-Out | Nest-In | Nest-Out |
| #GLS | #08^1^ | #08^1^ | #08^2^ | #08^2^ | #09^1^ | #09^1^ | #09^2^ | #09^2^ |
|  | 8 | 16 |  |  |  |  |  |  |
|  | 7 | 16 |  |  |  |  |  |  |
|  | 5 | 17 |  |  |  |  |  |  |
|  | 3 | 15 |  |  |  |  |  |  |
|  | 9 | 15 |  |  |  |  |  |  |
|  | 9 | 15 |  |  |  |  |  |  |
|  | 8 | 16 |  |  |  |  |  |  |
|  | 6 | 18 |  |  |  |  |  |  |
|  | 5 | 19 |  |  |  |  |  |  |
| Mean (h) | 10 | 18 | 10 | 25 | 9 | 16 | 10 | 17 |
| Total (h) | 570 | 1079 | 466 | 1181 | 433 | 721 | 429 | 768 |
| Mean (hd^-1^) | 8 | 16 | 7 | 17 | 9 | 15 | 9 | 15 |


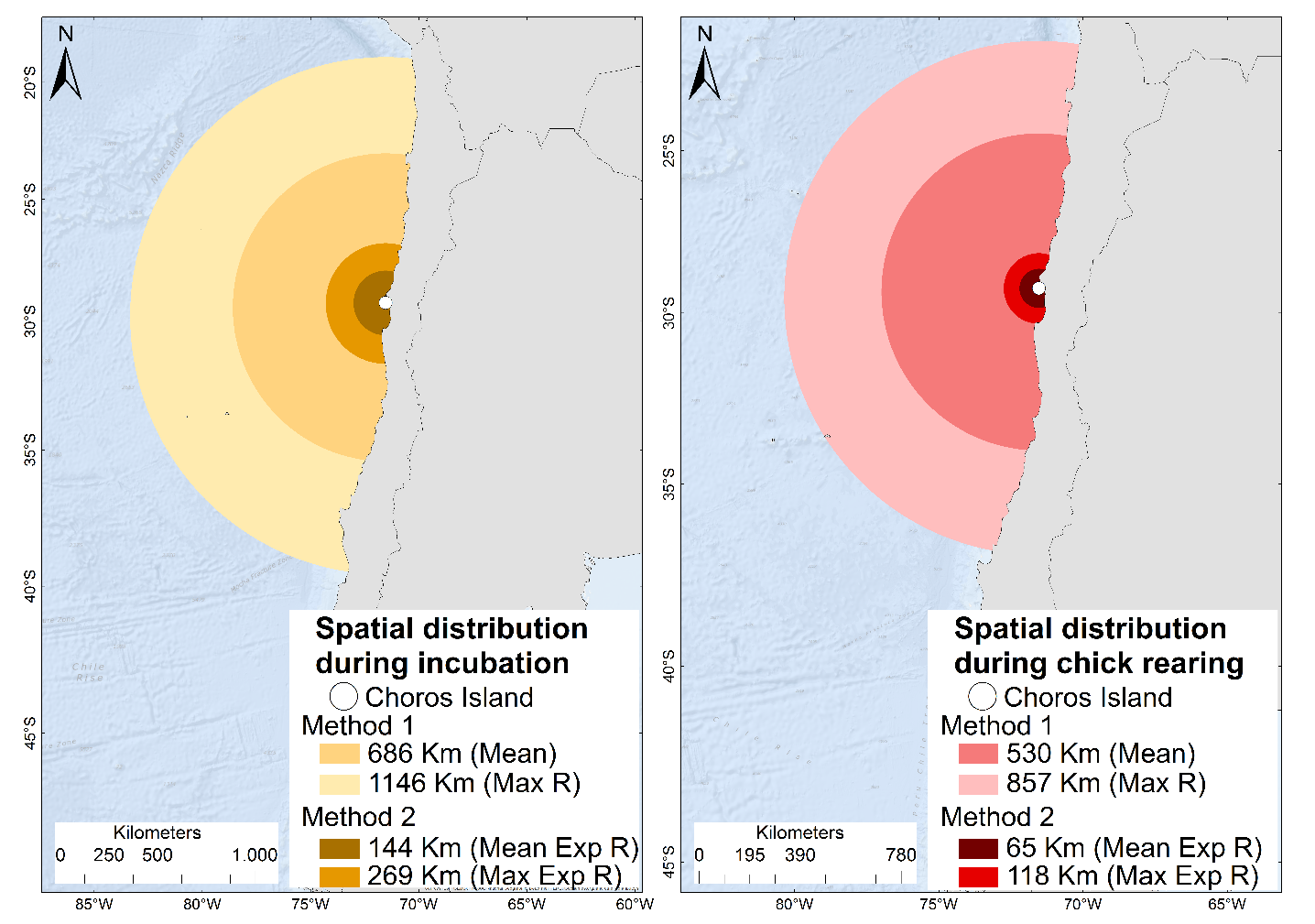


**Fig. S1** At-sea spatial distribution of Peruvian diving-petrels during breeding (incubation, chick rearing) season are represented by the influence zones (home distance values). For method 1 (home distance values from Actave.net program), the mean and the maximum range (Max R) are showed by darker and lighter tone contours, respectively. For method 2 (home distance corrected values), the mean expected range (Mean Exp R) and the maximum expected range (Max Exp R) are represented by darker and lighter tone contours, respectively.


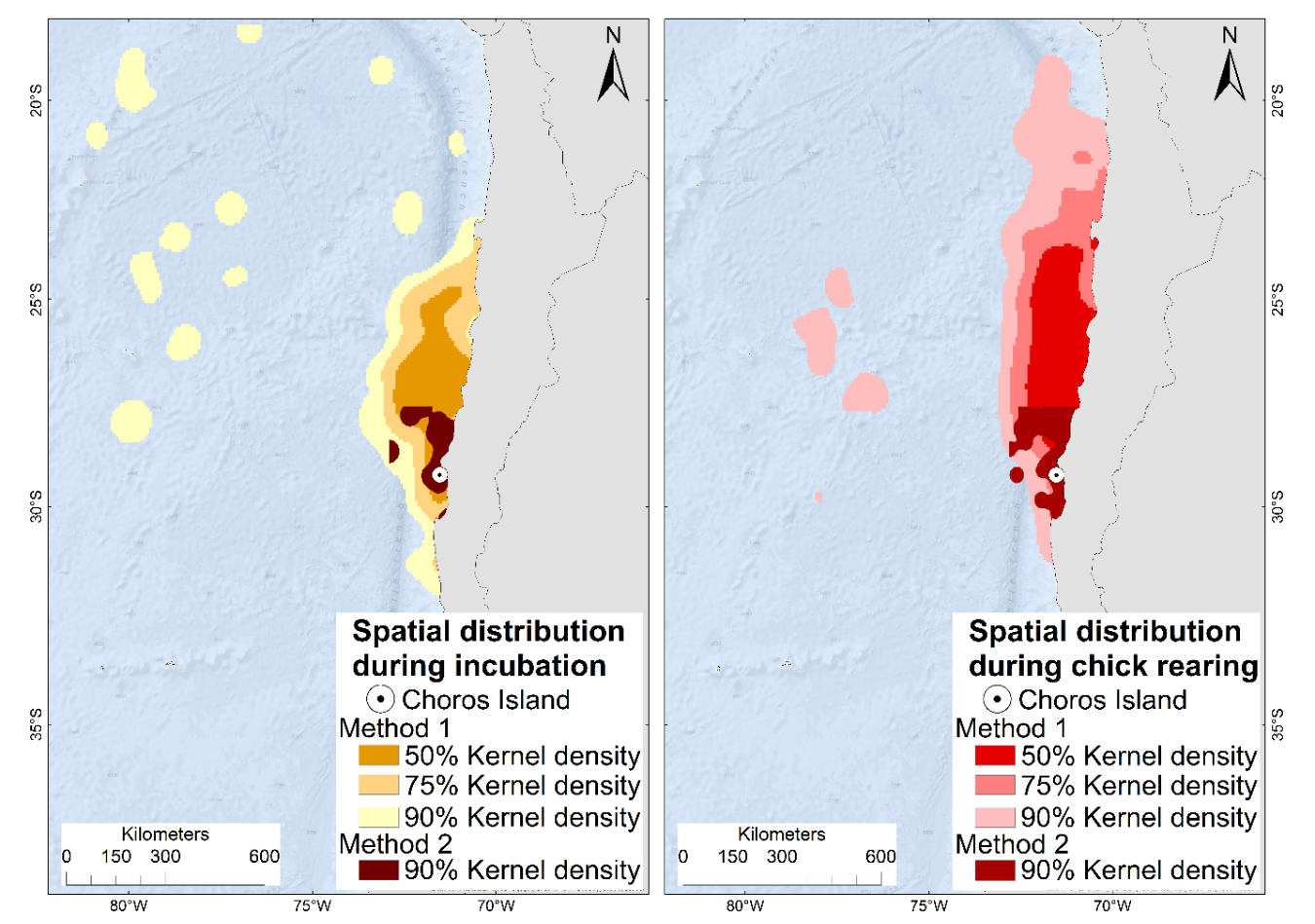


**Fig. S2** At-sea spatial distribution of Peruvian diving-petrels during breeding (incubation, chick rearing) season represented by kernel density areas. For method 1, 50%, 75% and 90% kernel density areas are represented from darker to lighter tone contours, respectively. For method 2, 90% kernel density area is represented.
